# Hemizygous loss of helicases promotes genomic instability and cancer development

**DOI:** 10.1126/sciadv.adv4540

**Published:** 2026-02-20

**Authors:** Karolin Voßgröne, Francesco Favero, Krushanka Kashyap, Francisco G. Rodríguez-González, André V. Olsen, Xin Li, Balca R. Mardin, Joachim Weischenfeldt, Claus S. Sørensen

**Affiliations:** ^1^ Biotech Research and Innovation Centre, University of Copenhagen, Ole Maaløes Vej 5, Copenhagen 2200 N, Denmark.; ^2^ Finsen Laboratory, Copenhagen University Hospital–Rigshospitalet, Copenhagen, Denmark.; ^3^ BioMed X Institute (GmbH), Heidelberg, Germany.; ^4^ Charité–Universitätsmedizin Berlin, Berlin, Germany.

## Abstract

Cancer mutations perturb key processes, driving uncontrolled cell proliferation. With critical roles of enzymes in cell function and growth, we hypothesized that cancer driver mutations alter specific and recurrent enzymatic functions. Leveraging large pan-cancer genomic datasets and curated mutation catalogs, we identified frequent mutations in helicases, enzymes involved in nucleic acid unwinding and processing. Helicases emerged as the most commonly mutated cancer driver enzyme family, altered in two-thirds of all cancers. Functional screens and genomic analyses revealed that helicase dysfunctions contribute to genomic instability and faulty DNA repair. We observed a marked phenotype of Aquarius helicase (*AQR*), which was recurrently hemizygously deleted as an early clonal event in cancer genomes. These deletions were associated with high genomic instability and homologous recombination deficiency signatures. Furthermore, we found hemizygous loss to be a common tumor suppression mechanism among helicases, present in 35% of all cancers. Overall, our enzyme-family approach highlights helicases, including *AQR*, as key potential cancer drivers.

## Teaser

Losing one copy of helicase genes reduces genome protection, leading to DNA damage and higher risk of cancer development.

## INTRODUCTION

Cancer is fueled by driver mutations in genes that promote and orchestrate cancer development and progression. The mutated oncogenes and tumor suppressor genes (TSGs) have classically been identified on the basis of the recurrence of aberrations affecting the genes, with gain-of-function (GoF) mutations of oncogenes and loss-of-function (LoF) mutations of TSGs. Cancer drivers have been challenging to identify due to their context dependence, both in terms of tissue type, cell of origin, and dependence on dysregulated pathways and other cancer genes. Statistical concepts and approaches have been developed to identify recurrently mutated genes within and among different cancer types (*1*, *2*) and enrichment of mutations in specific pathways (*3*, *4*). Despite advances, genetic alterations contributing to tumor development and progression are not well characterized for a substantial number of cancers. Current approaches seek to identify genes or loci that are more frequently mutated than expected by chance but do generally not consider a priori the function or activity of the affected genes.

An orthogonal approach is to harness information on the molecular function and activity of the mutated protein. A key function is enzymatic activity, such as phosphorylation and dephosphorylation of proteins by kinases and phosphatases, respectively. Enzymatic activities catalyze thousands of biochemical reactions in the cell (*5*). Specifically, biochemical classification of enzymes is based on common biochemical activity rather than sequence similarity. Accordingly, sequences within an enzyme class are often highly divergent; thus, the term families is used for sequence-related enzyme groups within a class (*6*). A number of enzyme families are associated with many cancer drivers such as kinases and phosphatases (*7*, *8*); however, it remains to be determined whether additional enzyme families aggregate cancer drivers and whether this could be harnessed further.

To address this gap in cancer knowledge, we develop an enzyme-family driver identification approach that combines information of enzyme-wide molecular activities and mutational frequencies. This pinpoints cancer driver functions toward enzyme types and prompts unbiased genotype-phenotype categorization and characterization. Here, we carried out an enzyme family-centric approach based on genomic data from 5844 patients with cancer. We identify an unexpectedly high rate of mutations across various cancer types in helicases and nucleases, enzymes involved in unwinding and processing of nucleic acids. Using a high-throughput functional phenotype screening approach, we uncover a major biological role for helicases in genome maintenance. Among this family of enzymes, we find and functionally characterize a particular notable phenotype for *AQR* and demonstrate that hemizygous loss of AQR is associated with distinct structural variant (SV) and mutation signatures in cancer genomes. In summary, the enzyme-family approach uncovers helicases as commonly mutated in cancer, points to a hemizygous driver mechanism particularly abundant in helicases, and provides a putative therapeutic option in tumors with hemizygous AQR loss.

## RESULTS

### Pan-cancer mutation enrichment analysis reveals helicases as a recurrently mutated enzyme family

**Fig. 1.**
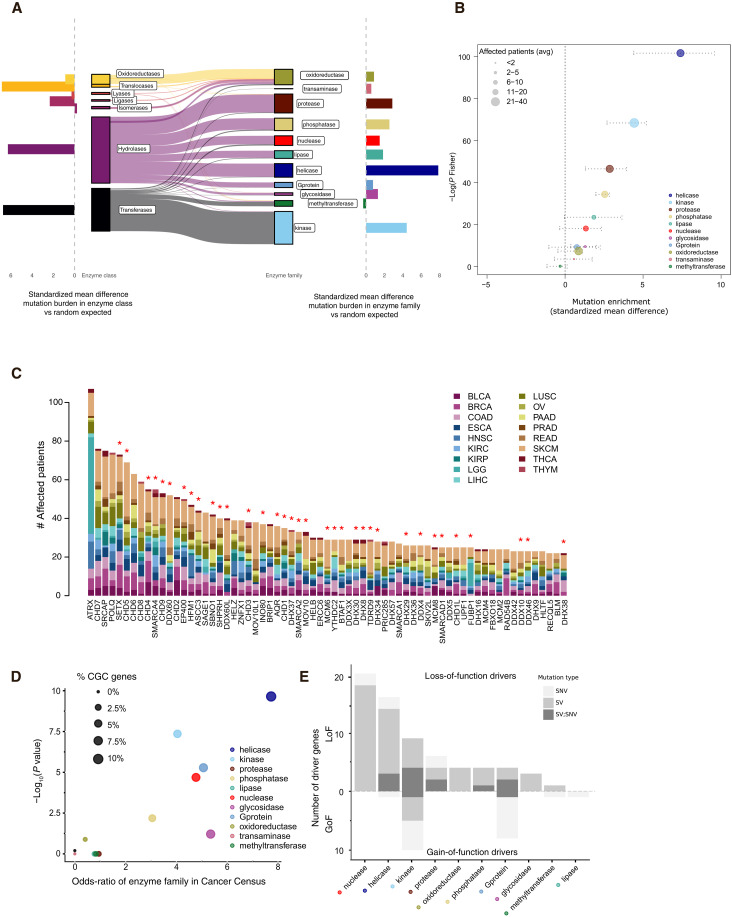
Cancer genomic analyses identify helicases as a recurrently mutated enzyme family. (**A**) Enzyme classes partitioned as UniProtKB first-level annotation (left) and PantherDB annotation for enzyme families (right). The horizontal bar plots on the left and right sides correspond to the average effect size. (**B**) Pan-cancer mutation enrichment significance by enzyme families; the *y* axis corresponds to the Fisher combined *P* value (−log) of 17 cancer studies from TCGA and METABRIC, and the *x* axis corresponds to the average effect size (colored points) computed as the mean difference of each enzyme family to a permuted background set. The range of effect size across all studied cancer types is represented as horizontal dotted lines. (**C**) Occurrence of somatic mutations in helicases: the most frequently mutated helicase genes ordered by occurrence. Each column corresponds to a gene, the colors in each column identify different tumor types, and the height of a column corresponds to the number of patients across 17 different tumor type studies (legend) carrying a mutation in the corresponding gene. Red asterisk on top of the bars denotes a significant GISTIC copy number loss peak. (**D**) Driver gene enrichment analysis for the 11 enzyme families. The CGC catalog from COSMIC (version 95) was used to test significant enrichment for known driver genes in each enzyme family. The significance level (−log *P* value, *y* axis) versus enrichment of CGC genes in each enzyme family (odds ratio, *x* axis). The dot size is proportional to the fraction of genes in the enzyme family present in the CGC. (**E**) For each enzyme family (*x* axis), the number of CGC driver genes classified as LoF (*y* axis, top bar plot) and GoF (*y* axis, bottom bar plot) separated by the type of driver mutation involving SNVs (light gray), SVs (medium gray), or both SNVs and SVs (dark gray).

To enable a comprehensive analysis of mutations in enzyme families, we first categorized genes belonging to the seven main enzyme classes: oxidoreductases, transferases, hydrolases, lyases, isomerases, ligases, and translocases (*9*). We analyzed the pan-cancer mutation burden for all genes in each enzyme class using publicly available genomic data generated by the TCGA Research Network (https://cancer.gov/tcga) and METABRIC (*10*) across 18 different cancer types. By comparing the observed number of mutations in each class to the expected distribution of mutations, we found an increased mutational frequency in translocases, hydrolases, and transferases (effect size = 6.8, 6.3, and 6.7; *P* = 7.7 × 10^−39^, 1.5 × 10^−37^, and 2.3 × 10^−36^, respectively, Fisher’s combined probability; Fig. 1A). This prompted us to investigate the associated enzyme families, a further division of the main enzyme classes characterized by amino acid sequence and structural similarities. To this end, we identified genes belonging to 11 enzyme families including 409 oxidoreductases, 25 glycosidases, 159 G proteins, 365 kinases, 75 lipases, 73 methyltransferases, 173 phosphatases, 267 proteases, 18 transaminases, 162 helicases, and 156 nucleases (*11*). Oxygenases were removed from further analyses due to a high redundancy with oxidoreductase enzyme family and a low number of unique enzymes. Adjusting for the number and size of genes in each family, we found cancer-related kinase, protease, and phosphatase enzyme families to be significantly mutated across all cancer types (*P* = 1.9 × 10^−30^, 6 × 10^−21^, and 1.1 × 10^−15^, respectively, 10,000 permutations, Fisher’s combined probability). Unexpectedly, we found helicases to be the most frequently mutated enzyme family (mutation burden corrected for gene length, effect size = 7.4, *P* = 7.1 × 10^−45^, Fisher’s combined probability; Fig. 1, A and B). At the individual gene level, we found cancer-associated genes such as *ATRX*, *POLQ*, and *SMARCA4* to be frequently mutated helicases across different cancers (Fig. 1C). We note that gene expression levels did not appear to differentially affect mutation rates within the enzyme families (table S1). Moreover, helicases displayed the most stable expression level across different cancer types (fig. S1A).

To explore whether and to what extent these enzyme families were enriched for driver genes, we next performed an unbiased enrichment analysis to compare the observed versus expected number COSMIC Cancer Gene Census (CGC) catalog of known driver genes (version 95) in each enzyme family. In agreement with our pan-cancer mutation burden analysis, helicases showed the strongest enrichment among CGC genes (odds ratio = 7.7, *P* < 1 × 10^−10^, Fisher’s exact test, false discovery rate–corrected *P* value; Fig. 1D), with 18 of 145 helicases listed as CGC, followed by kinases, phosphatases, and G proteins. Helicase CGC genes were associated with escaping programmed cell death and genome instability (fig. S1B and table S2) including *ATRX*, *BLM*, *DICER1*, *ERCC2*, and *RECQL4*.

To gain more insights into the potential cancer driver mechanism for the different enzyme families in general and helicases in particular, we next used whole-genome sequencing (WGS)–based mutational analysis from the pan-cancer analysis of whole genomes (PCAWG) consortium, spanning more than 2600 cancer genomes (*12*). We used the PCAWG-curated list of driver genes (*1*, *12*), and performed an unbiased analysis for each enzyme family. We separated the driver mutation by the type of mutation [single-nucleotide variants (SNVs), SVs including copy number alterations, and whether they were LoF or GoF mutations]. In agreement with the CGC analysis, we found no or very few driver genes in transaminases, lipases, and methyltransferases (Fig. 1E). Notably, nucleases and helicases were almost exclusively associated with LoF mutations, primarily driven by SVs (gray and dark gray bars). In total, 20 of 20 (100%) nucleases and 16 of 17 (94%) helicases were exclusively LoF driver mutations (Fig. 1E). Given the importance of LoF alterations, we next used the larger TCGA dataset to perform a copy number recurrence analysis using GISTIC (*13*), focusing on genes with copy number losses. We found 35 of the 96 (36%) most frequently mutated helicases and nucleases to be associated with a significant GISTIC loss (*P* = 2.8 × 10^−12^ and 4.15 × 10^−16^ for helicases and nucleases, respectively, Fisher’s combined probability; table S3), with 31 (89%) of these being significant in two or more cancer types (Fig. 1C, red asterisks). In comparison, frequently mutated enzyme families such as proteases, phosphatases and G protein enzymes were more frequently associated with GISTIC amplification peaks (table S3). Analysis of the dN/dS ratios across enzyme families and cancers based on the TCGA dataset did not indicate frequent occurrence of truncating mutations in helicases and nucleases (fig. S1C). At the individual gene level, we found nuclease TSGs such as *FANCM* and *DICER* to be frequently mutated in a variety of cancers (fig. S1D) (*14*–*16*).

Last, we asked whether specific cancer types were enriched for alterations in helicases and nucleases. Across 18 different cancer types (9660 patients), we found breast cancer (BRCA) to be highly enriched, with 151 of 304 (50%) helicase and nuclease genes to be mutated in at least two patients, compared to an average of 54 (18%) helicase and nuclease genes for the other 16 non–skin cancer types (*P* < 1 × 10^−4^, 10,000 permutations; fig. S1E). Pathway analysis showed that the helicase and nuclease genes mutated in this cancer subtype were enriched for DNA repair functions, unwinding of DNA, and DNA strand elongation (fig. S1F), suggesting deficiencies in genome maintenance.

In summary, we find that helicases and nucleases represent highly mutated enzyme families with the highest proportion of known cancer-driver genes, almost all of which are LoF associated with somatic SVs.

### Genetic screen identifies LoF helicases and nucleases associated with genomic instability

**Fig. 2.**
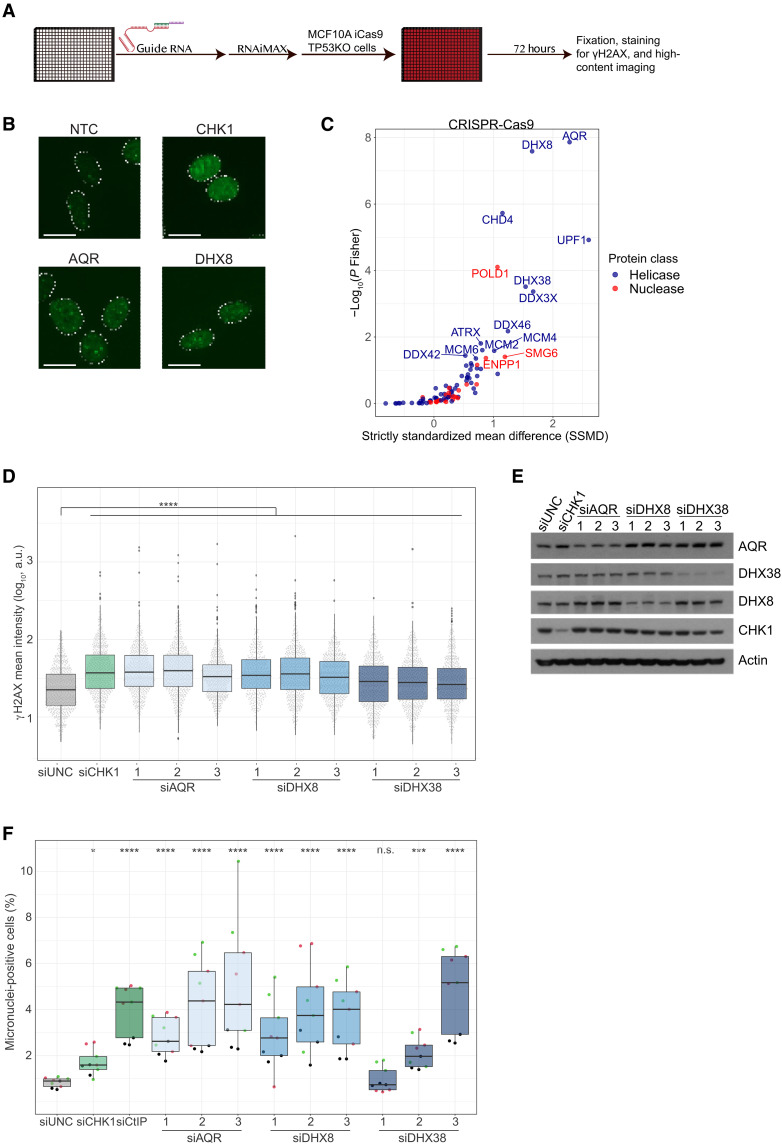
Phenotype screens identify cancer-associated genome maintenance helicases and nucleases. (**A**) Genome instability screen scheme. Guide RNA complexes were reversely transfected into MCF10A iCas9 p53KO, and cells were fixed 3 days posttransfection. Samples were stained for DAPI and γH2AX. (**B**) Representative images of γH2AX. Outline indicates DAPI staining. Scale bars, 20 μm. (**C**) MCF10A iCas9 p53KO CRISPR screen results, performed as shown in (A). On the *x* axis, the effect size is plotted as the SSMD in frequency of γH2AX-positive cells. The *y* axis corresponds to the Fisher combined *P* value from three biological replicates. Colored by enzyme family and labeled dots represent significantly scoring genes (*P* Fisher < 0.05). (**D**) siRNA screen validation of AQR, DHX8, and DHX38-depleted MCF10A iCas9 p53KO cells. Timing as in (A), depicting the γH2AX mean intensity on the *y* axis. Representation of one of three biological replicates. *n* = 600, *n* = 200 per technical replicate. ****P < 0.0001. a.u., arbitrary units. (**E**) Immunoblot analysis of siRNA screen validation samples shown in (D). Actin was used as a loading control. (**F**) Frequency of micronuclei-positive U-2 OS cells of the same samples as shown in fig. S2 [(D) to (F)]. Representation of three biological replicates, each with three technical replicates. Point colors indicate samples belonging to the same biological replicate. *n* = 9. n.s., not significant.

We were motivated by our cancer genomic findings to investigate potential roles of helicases and nucleases in genome maintenance through LoF screening. Therefore, we designed small interfering RNA (siRNA) and CRISPR libraries targeting the 96 most frequently mutated helicase and nuclease genes (table S4). We performed image-based phenotypic arrayed screens using siRNA in the osteosarcoma cell line U-2 OS that allows straightforward detection of direct genome instability evident as micronuclei formation (*17*, *18*). Furthermore, we carried out siRNA and CRISPR screens in nontransformed breast epithelial MCF10A iCas9 p53KO cell line (*19*, *20*). Both RNA interference (RNAi) and CRISPR-Cas9 screens were performed in 384-well plate format, targeting each gene individually with three different siRNAs or guide RNAs, respectively. Deficiency in genome maintenance was analyzed 3 days posttransfection by staining for γH2AX and examining micronuclei formation, as established markers of DNA damage (*21*, *22*) (Fig. 2, A and B). In total, we found 9 of 96 tested genes to be significantly associated with genome maintenance in both the siRNA and the CRISPR screen in the MCF10A iCas9 p53KO model (*P* < 0.05, Fisher’s combined *P* value; Fig. 2C; fig. S2, A to C; and table S4). *Aquarius* (*AQR*) was the top-scoring gene followed by the *DEAH-box helicase 8* (*DHX8*) in both the siRNA and CRISPR screens. We noted that the top-scoring genes *AQR*, *DHX8*, and *DEAH-box helicase 38* (*DHX38*) were all RNA helicases belonging to the superfamily II (SF2) type. They were validated in independent experiments (Fig. 2, D to F, and fig. S2, D to F). In conclusion, we identified the recurrently mutated RNA helicase genes *AQR*, *DHX8*, and *DHX38* as likely genome maintenance factors. RNA and DNA helicases differ, both in substrate and function: DNA helicases unwind double-stranded DNA (dsDNA) during replication, repair, and recombination. RNA helicases remodel RNA structures or RNA-protein complexes. RNA helicases also unwind DNA-RNA hybrids to suppress R-loop formation. Notably, *AQR* has previously been associated with increased genome instability (*23*, *24*) but neither of these three genes were linked with tumor suppression. In line with our findings from the enzyme family-centric analysis, our data showed an enrichment of helicase genes with marked genome maintenance function compared to nuclease genes (fig. S2G).

### AQR hemizygous tumors are associated with homologous recombination deficiency and genome instability

**Fig. 3.**
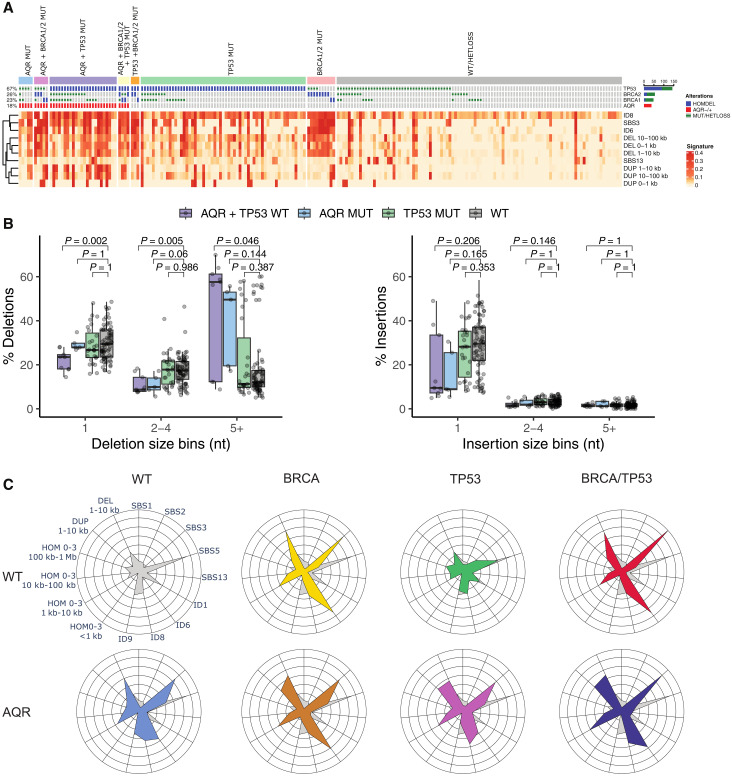
Hemizygous loss of AQR is linked to genome instability and deregulated DNA repair pathways including homologous recombination. (**A**) Oncoprint showing the PCAWG breast cohort stratified by hemizygous *AQR* loss, as well as *TP53*, *BRCA1*, and *BRCA2* homozygous loss. Each column represents a donor, and displays relative exposures of indels signatures ID8 and ID6, mutational signatures SBS3 and SBS13, as well as small duplication and small deletion patterns between 0 to 100 kb. (**B**) Deletions (left) and insertions (right) grouped by 1-, 2-, to 4-nt or bigger than 5-nt indels in *AQR* heterozygous loss samples (AQR MUT) *AQR* heterozygous loss and TP53 homozygous loss double-mutant (AQR+TP53 MUT) and AQR wt and TP53 wt (WT). (**C**) Radar plot showing the mutational signature exposure of breast cancer samples with heterozygous *AQR* loss (AQR, bottom row) and comparing to cancers without BRCA or TP53 (WT), with *BRCA1* and/or *BRCA2* homozygous loss (BRCA), *TP53* homozygous loss (TP53), or both BRCA1/2 and TP53 homozygous loss (BRCA/TP53).

Next, we pursued a pan-cancer analysis of our top-scoring gene *AQR* to further explore its role in genomic instability. Our initial cancer genomic analysis identified recurrent copy number losses spanning *AQR* (Fig. 1C). In agreement, we found frequent heterozygous copy number loss at *AQR* (fig. S3A), which was associated with increased genomic instability (fig. S3B, *P* < 0.0001) to a level comparable to that of tumors with heterozygous loss of *TP53*. In contrast to *TP53* and *BRCA2*, we found a significant reduction of homozygous *AQR* loss (fig. S3B, *P* = 0.016, Fisher’s exact test), suggesting haploinsufficiency. Motivated by the link between *AQR* loss and copy number genomic instability in breast cancer from our pan-cancer analysis and mammary cell lines (Figs. 1C and 2 and fig. S1, D and E), we next used the PCAWG WGS resource to explore whether breast cancer samples with *AQR* loss displayed particular patterns of mutational signatures. We compared breast cancer samples with heterozygous loss of *AQR* (*n* = 38) to cancers with homozygous loss of *BRCA1* (*n* = 7), *BRCA2* (*n* = 15), or *TP53* (*n* = 90). Regressing different mutational signatures on the mutation status of these genes, we found an expected association between *BRCA1* (*P* = 6.48 × 10^−5^, yellow cross) and *BRCA2* (*P* = 2.18 × 10^−5^, red cross) and the mutational signature SBS3 (“BRCAness” signature; Fig. 3A and figs. S4 and S5), which has been found to be elevated in tumors with homologous recombination deficiency (HRD) (*25*). Although cancers with heterozygous *AQR* loss or *TP53* homozygous loss alone (*P* = 0.012, blue square and *P* = 0.005, green cross, respectively) were associated with elevated SBS3, we found the strongest association in samples with both *AQR* and *TP53* loss (*P* = 1.75 × 10^−8^, purple circle; Fig. 3A and figs. S4 and S5). Our analysis confirmed the previously reported association between *BRCA2* homozygous loss and ID6 (*P* = 4.77 × 10^−12^; Fig. 3A and figs. S4 and S5) (*26*, *27*), an indel signature characterized by deletions 5 base pairs (bp) or longer with flanking microhomology. Consistent with our SBS3 signature, we again found *AQR*/*TP53* double-mutant cancer samples to have elevated levels of ID6 (*P* = 1.92 × 10^−5^) but even more so for ID8 (*P* = 2.49 × 10^−10^). ID8 is characterized by deletions of 5 bp or more and minimal microhomology and is linked with nonhomologous end joining (NHEJ) (*27*). We next investigated larger deletions and duplications in size ranges of 0 to 1 kb, 1 to 10 kb, 10 to 100 kb, and 100 kb to 1 Mb, which again revealed the expected association between *BRCA2* and short deletions as well as between *BRCA1* and short- to medium-sized duplications (Fig. 3A and figs. S4 and S5) (*28*). Although *AQR*/*TP53* double-mutant cancers displayed increased levels of short deletions (1 to 10 kb, *P* = 1.51 × 10^−3^; Fig. 3A and figs. S4 and S5), the double-mutant cancers were associated with an even more notable accumulation of short- and medium-sized duplications (1 to 10 kb, *P* = 9.70 × 10^−10^ and 10 to 100 kb, *P* = 1.45 × 10^−7^; Fig. 3A and figs. S4 and S5) (*28*). We also found deletions and duplications in *AQR*/*TP53* double-mutant cancers to have short flanking homologies (0 to 3 bp; fig. S4). Our analysis identified strong co-occurrence of mutational signatures, including ID8, SBS3, ID6, short deletions, and duplications in *AQR*/*TP53* double-mutant cancers (Fig. 3B, purple bar) but not signatures associated with other mutational processes, such as APOBEC (Fig. 3B, SBS13). *TP53* homozygous loss cancers—without *AQR* heterozygous loss—displayed insignificant associations with the HR- and NHEJ-related signatures (*P* > 0.05; Fig. 3, A and B, and fig. S3). In support of a tumor-promoting effect, we found significant co-occurrence of *AQR* and *TP53* (*P* < 2.22 × 10^−16^), whereas *BRCA1* and *BRCA2* were much less likely to co-occur with *AQR* loss (fig. S6, A to C). We performed clonality analysis to assess the order of events in *AQR*/*TP53* double-mutant cancers, which showed that both events were early clonal events (no difference in clonality, *P* = 0.15, Fisher’s exact test; fig. S7). This suggests that co-occurring *TP53* and *AQR* mutations are early clonal events, likely under positive selection.

Motivated by the strong association with short deletions (ID8), we compared the proportions of deletions and insertions of 1 and 2 to 4 bp and deletions of length 5 or more (5+; Fig. 3C). We found no difference for cancers with homozygous *TP53* loss alone. Heterozygous *AQR* loss, on the other hand, was associated with a significantly higher proportion of 5+ deletions specifically (Fig. 3C, left), which was exacerbated in double-mutant *AQR*/*TP53* cancers.

Summarizing the different mutational signatures and comparing cancers without *AQR*, *TP53*, *BRCA1*, and *BRCA2* (“WT” in Fig. 3D), we found *AQR* private-mutated cancers to display elevated levels of SBS3, ID6, ID8, and 1- to 10-kb deletions (Fig. 3D, blue radar plot), further increased in *AQR*/*TP53* double-mutant cancers (Fig. 3D, purple radar). *TP53* private mutations did not lead to elevated levels of the investigated signatures compared with wild type (WT) (Fig. 3D, green radar). *BRCA1* or *BRCA2* mutated cancers (Fig. 3D, “BRCA”, yellow radar) was associated with SBS3 and ID6, which was not notably affected by co-mutations with *AQR* (Fig. 3D, brown radar) or *TP53* (Fig. 3D, red radar). These findings suggest that homozygous loss of *TP53* potentiates cancers with *AQR* loss, leading to a more extreme genomic instability phenotype, characterized by elevated levels of both HR- and NHEJ-related signatures.

To further explore the functional role of AQR in genome maintenance and HR, we generated an inducible siRNA-resistant AQR–green fluorescent protein (GFP) system in MCF10A cells. We first used γH2AX as a marker for DNA damage to measure the impact of *AQR* depletion in MCF10A cells. We found an increase in γH2AX signal upon *AQR* knockdown, which we could rescue using the inducible AQR-GFP system (fig. S8, A and B). Next, we assessed HR efficiency upon knocking down AQR using the Direct Repeat GFP (DR-GFP) reporter system cells, which showed almost twofold reduced HR efficiency following AQR depletion (fig. S8, B and C), in line with a previous report (*29*). The presence of RNA:DNA hybrids, also called R-loops, can affect HR repair (*30*, *31*), and AQR has been proposed to be able to resolve R-loops (*24*). For this reason, we tested whether overexpression of RNase H1, an enzyme that degrades the RNA moiety in R-loops, can rescue DNA damage accumulation. Using knockdown of senataxin (SETX) as a positive control, we found that RNase H1 overexpression in AQR-depleted cells reduced levels of the γH2AX marker (fig. S8, E and F). This suggests that AQR maintains genome stability in part through R-loop homeostasis.

### AQR haploinsufficiency causes S phase–specific damage and sensitivity to PARP inhibitor

**Fig. 4.**
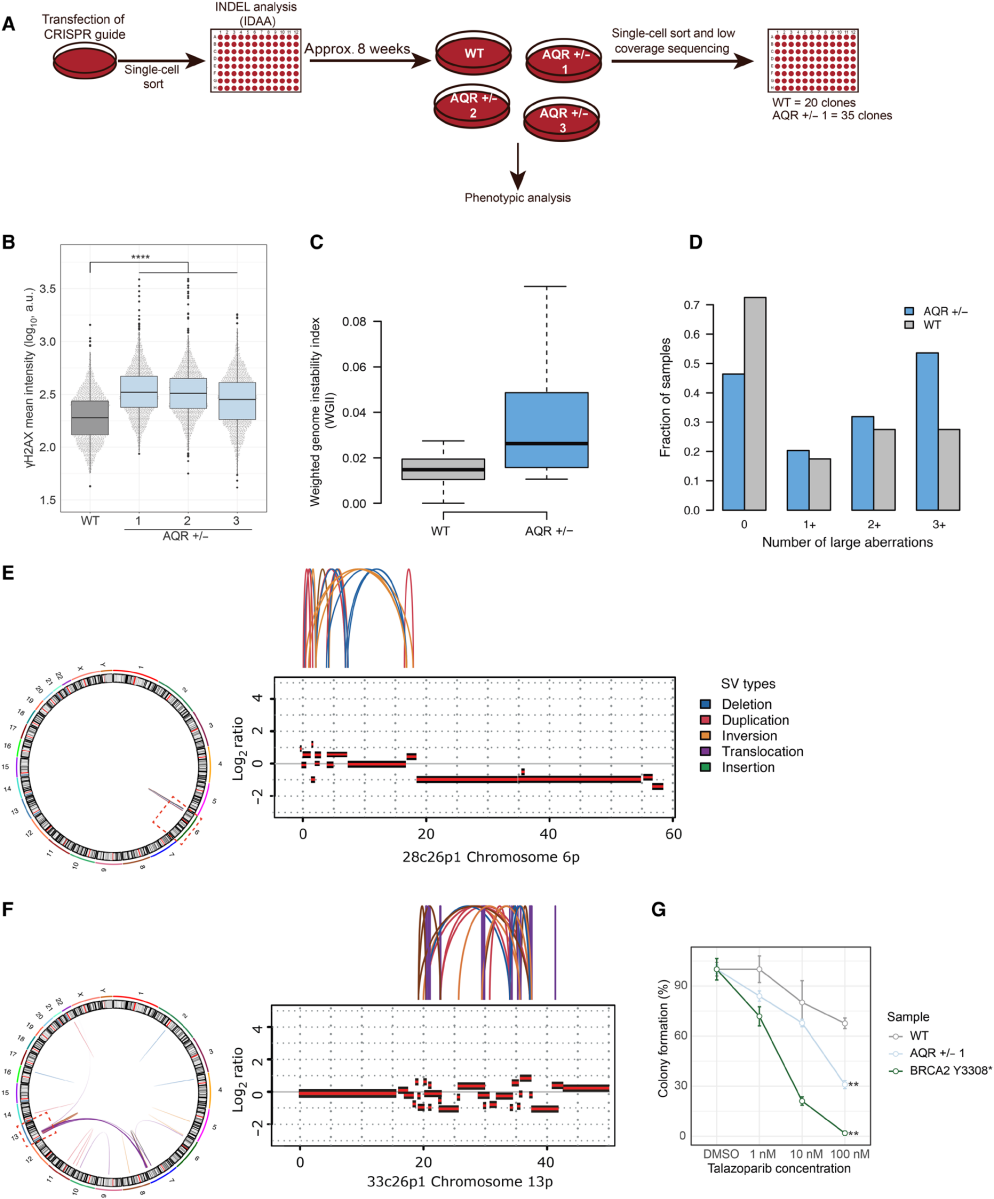
AQR heterozygosity causes increased genome instability and sensitivity to PARP inhibition in a cell line model. (**A**) Schematic presentation of the generation and culturing of *AQR* heterozygous MCF10A iCas9 p53KO cells. (**B**) Analysis of the γH2AX mean intensity (*y* axis) of WT and *AQR* heterozygous MCF10A iCas9 p53KO cells. Representation of one of three biological replicates, *n* = 1500 cells per sample, *n* = 500 per technical replicate. ****P < 0.0001 compared to *AQR* WT cells. (**C**) WGII of WT clones (gray) and *AQR* hemizygous depleted clones (blue), *P* = 7.8 × 10^−225^. (**D**) Paired bar plots of WT clones (gray) and *AQR* hemizygous depleted clones (blue) showing the fraction of samples without any copy number alteration (first column from the left), at least 1, 2, or 3 copy number alterations (second, third, and last column from the left, respectively). (**E** and **F**) Chromosomes 6p and 13p of two *AQR* hemizygous depleted clone with clone-specific complex SVs. Circos plot representing the genome-wide scale of SVs (left) and genomic coverage (right) with arches representing the edges of SV resulting after the rearrangements. The bottom panel shows the log_2_ ratio of the coverage of a wt clone versus the coverage of the mutated clone. Positive values represent copy number gains, and the negative value represents copy number losses. (**G**) Colony formation of *AQR* WT or *AQR* heterozygous clone 1 MCF10A iCas9 p53KO and *BRCA2* Y3308* MCF10A iCas9 cells treated for 2 weeks with the indicated doses of talazoparib. Representation of technical triplicates within one biological replicate. **P < 0.01, compared to the WT control.

Our findings implicate a haploinsufficient tumor suppressor role for AQR, mediated via genome maintenance. To further explore this, we generated *AQR* heterozygous MCF10A iCas9 p53KO cells using CRISPR-Cas9 gene editing (Fig. 4A). We were only able to obtain hemizygous but no homozygous knockout clones, in agreement with the essential role of AQR in cell survival. *AQR* hemizygosity led to a decrease in AQR protein levels (fig. S8A), and the *AQR* hemizygous cells showed increased γH2AX levels compared to *AQR* diploid cells (Fig. 4B) but no change in cell cycle, as assessed by total 4′,6-diamidino-2-phenylindole (DAPI) intensity and mean 5-ethynyl -2′-deoxyuridine (EdU) intensity (fig. S9). To further evaluate the impact of *AQR* hemizygosity, we performed low-coverage (0.5 to 1x coverage) WGS of 20 *AQR* hemizygous and 20 *AQR* diploid clones, followed by copy number analysis. The *AQR* diploid control cells exhibited low but detectable levels of genomic instability, likely due to the *TP53* null background and residual off-target effects of the inducible Cas9. In contrast, we found a significant increase in whole-genome instability in the hemizygous *AQR* clones (*P* = 7.8 × 10^−225^; Fig. 4C). Most *AQR* hemizygous clones had three or more large genomic aberrations, compared to ~25% of control clones (Fig. 4D). Although we found no evidence of high copy number burden in any of the WT clones from our shallow WGS analysis, we performed deep WGS of two hemizygous *AQR* clones with a high copy number burden. This demonstrated complex SVs in both the deeply sequenced clones: one with localized complex SVs at chromosome 6p (28c26p1; Fig. 4E) and the other clone (33c26p1; Fig. 4F) with a locus on chromosome 13 showing highly complex SVs involving a series of small copy number oscillations, reminiscent of the HRD patterns observed in our cancer genomic analysis (Fig. 3). The complex rearrangements at chromosome 13 were connected with additional complex SVs involving chromosomes 6, 7, and X (Fig. 4F), suggesting the occurrence of multiple simultaneous double-strand breaks (DSBs) on these chromosomes, followed by illegitimate religation, creating a series of highly connected rearrangements.

HRD is typically associated with poly(ADP-ribose) polymerase (PARP) inhibitor sensitivity. Thus, we sought to test whether *AQR* haploinsufficiency sensitizes cells to PARP inhibition, potentially posing treatment options. We used colony formation assay using *AQR* WT MCF10A iCas9 p53KO, *AQR* hemizygous, and a positive control cell line, the MCF10A *BRCA2* homozygous Y3308* mutant, which is classified as pathogenic by ClinVar. The *AQR* hemizygous cells showed sensitivity toward the PARP inhibitor talazoparib relative to diploid *AQR* cells (Fig. 4G). These results support a role of AQR in HR repair and are in line with the identification of several mutational events associated with HRD in *AQR* hemizygous tumors.

### Helicases have a high prevalence of hemizygous cancer drivers

**Fig. 5.**
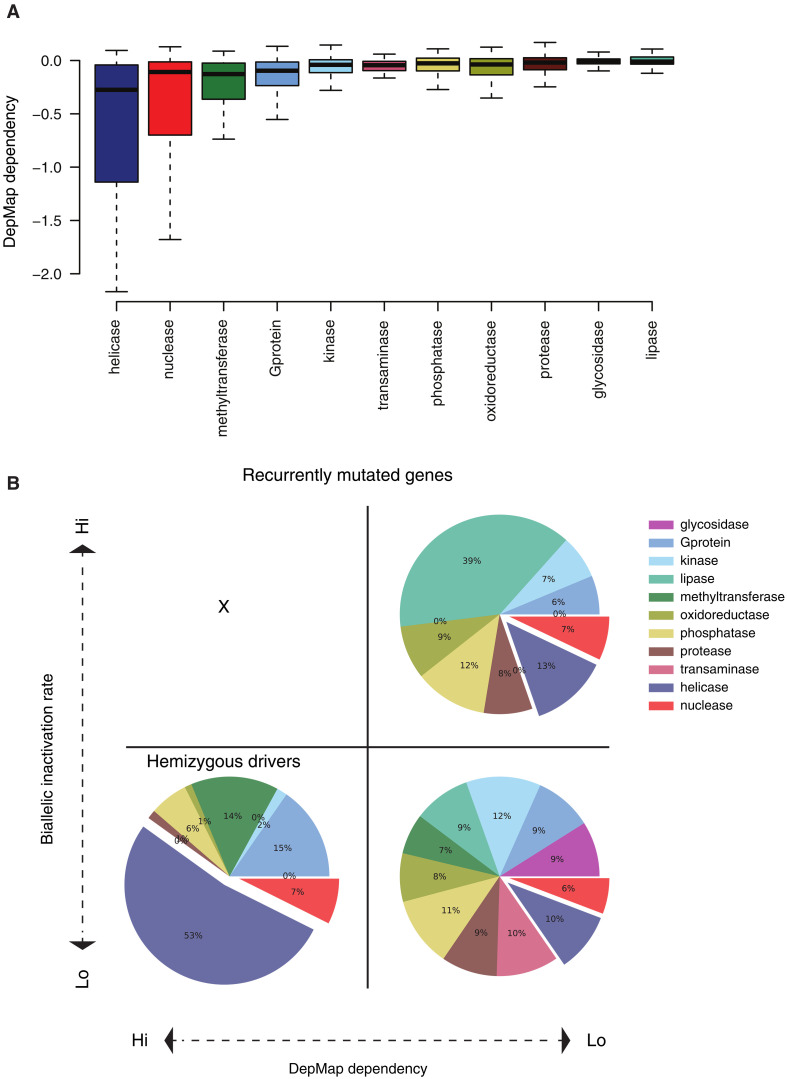
Helicases are enriched for hemizygous driver alterations in DepMap dependence genes. (**A**) Helicase enzymes are highly enriched for dependency genes. DepMap dependency score for the genes in each of the 11 enzyme families based on the DepMap CRISPR 22Q4 screen. (**B**) Hemizygous drivers identified as recurrently mutated genes (at least three focal mutations or a pan-cancer focal recurrence of >0.1%), essentiality (dependency score < −1), and not found as biallelic inactivated (<0.5%).

Our pan-cancer analysis showed that helicases are (i) frequently mutated, (ii) almost exclusively TSGs, and (iii) frequently affected by SVs and copy number loss. Moreover, our results indicate that *AQR* has a hemizygous cancer driver role with homozygous lethality. In keeping with this, we found *AQR* to be a common essential gene in DepMap (*32*), a large-scale functional genomics profiling of cancer dependencies and essential genes. To explore whether recurrent mutations leading to hemizygosity of essential genes represent a broader group of cancer driver genes, we combined mutation recurrence from the PCAWG cohort with gene dependencies from the DepMap genome-wide screen. To this end, we found that helicases on average display higher essentiality (Fig. 5A, negative DepMap Dependency score represents the level of essentiality in the CRISPR screen), compared to the other enzyme families.

We next used a simple schema to classify hemizygous driver candidate genes, requiring gene dependence essentiality (DepMap dependency score < −1), recurrently mutated (requiring at least three focal somatic alterations and a focal mutations rate of >0.1% in the cohort), and strongly depleted for biallelic inactivation (biallelic rate < 0.1%). We separated genes and their associated enzyme family into four groups: (i) nonessential, hemizygous (Fig. 5B, bottom right); (ii) nonessential, biallelic inactivation (representing well-known TSGs such *TP53* and *PTEN*; Fig. 5B, top right); (iii) essential and biallelic inactivated, which as expected never occurred (Fig. 5B, top left); and (iv) essential, hemizygous (Fig. 5B, bottom left). We found, in total, 42 putative hemizygous cancer drivers, 23 of which were helicases including *AQR* (55%, *P* = 1 × 10^−12^, Fisher’s exact test; table S5). Helicases were underrepresented among nonrecurrently mutated genes (fig. S10A). In support of critical roles in nucleic acid maintenance and processing, the helicase hemizygous driver candidates were enriched for genes involved in DNA and RNA processes such as DNA replication, Spliceosome, and NHEJ (fig. S10B).

In summary, we identify a subset of putative hemizygously mutated TSGs highly enriched for helicases. This group is characterized by frequent heterozygous losses but lacks homozygous losses, a pattern corroborated by high essentiality and indicative of important cellular functions.

## DISCUSSION

Although proteins have multiple roles in cellular and organismal processes, cancer driver functions are frequently linked with their catalytic activities. We based our study on this notion, and with a focus on enzyme function, we uncovered substantial differences in cancer mutation rate across the 11 different enzyme families. Although prior work has focused especially on kinases as drivers in cancer, we found an unexpectedly high mutation rate in helicases, a family of enzymes involved in processes including transcription, replication, translation, and DNA repair. We demonstrate that helicases are a predominant family of cancer drivers and that somatic alterations affecting helicases are almost exclusively associated with LoF. In agreement, we found helicases to be frequently disrupted by SVs, including copy number losses and rarely acquiring truncating point mutations. This points to helicases resembling C class enzyme tumors (*33*), characterized by frequent copy number alterations. The frequent copy number alterations of helicases resulted in hemizygosity, which we suggest is predicated on haploinsufficiency and the common essential roles of a subset of helicases. Knudsen’s classical two-hit hypothesis has been used to guide the discovery of TSGs in cancer, but a large proportion of the cancer genome is undergoing recurrent hemizygous loss (*34*). Several TSGs have been associated with a haploinsufficient role in cancer, including *SMAD4*, *TP53*, *CDKN1B*, *NF1*, and *PTEN* (*34*–*36*). Notably, these haploinsufficient genes all display frequent biallelic inactivation, associated with a more severe cancer-driving mechanism (*12*, *37*, *38*). Here, we extend this concept and provide evidence for the presence of a subset of hemizygous essential TSGs, particularly frequent in helicases. Helicases displayed stable gene expression across different cancers (fig. S1A), suggesting that members of this enzyme family are involved in basic and common cellular processes across all major cancer types. This is further corroborated by the important role of helicases in essential processes involving the unwinding of nucleic acids. It is, therefore, tempting to speculate that the helicase protein levels are finely tuned to operate in these vital processes and therefore sublethal but vulnerable to reduced dosage upon hemizygous loss, leading to cell survival but with genomic instability under hemizygous conditions.

The nuclease family was also enriched for LoF cancer drivers, although mutation frequency was lower than for helicases. A number of helicases and nucleases play important roles in DNA repair and suppression of genome instability in cancer (*39*, *40*), and we show that they are almost exclusively undergoing genomic losses rather than gains in cancer genomes. This motivated us to pursue functional LoF screening of the most frequently mutated helicases and nucleases from our pan-cancer analysis, and it enabled us to extend, compare, and functionally test the cancer-promoting or cancer-suppressing function of multiple potential driver genes (*41*–*44*).

Among the 96 candidate helicases and nucleases, AQR suppression displayed a particularly strong phenotype associated with genome maintenance. In keeping with a hemizygous role, we leverage a large pan-cancer WGS dataset to find three notable features of AQR. First, *AQR* exhibits recurrent hemizygous but never homozygous loss. Second, cancer genomes with hemizygous *AQR* loss frequently co-occur with homozygous *TP53* loss, and we provide support that *TP53* potentiates the genomic consequences of AQR loss, including genomic signatures compatible with NHEJ and HRD in breast cancer genomes. Third, although based on a modest number of samples, clonogenic experiments with hemizygous loss of *AQR* result in similar complex somatic aberrations and it is associated with sensitivity toward PARP inhibitors. We note that *RAD51*, a gene involved in HRD and found to interact with BRCA1 and BRCA2 (*45*, *46*), is located ~6 Mb downstream of *AQR* and is frequently lost in conjunction with AQR losses. Loss of both *AQR* and *RAD51* may be additive in contributing to the observed HRD-related mutational signatures, but we find compelling support for an AQR-private role. First, our CRISPR and siRNA-based screens specifically target *AQR*, and loss of *AQR* was associated with a strong phenotype associated with genomic instability. Second, cloning of hemizygous CRISPR-engineered *AQR* cells led to an accumulation of somatic alterations including complex SVs. Last, one of the breast cancer samples with a strong signature exposure of both SBS3, ID6, ID8, and small deletions had a small genomic deletion that encompassed *AQR* but not *RAD51* (fig. S5B, second from the right in the “AQR MUT” group), supporting an *AQR*-specific phenotype.

In the *AQR* hemizygous tumor samples, genome instability and HRD-related signatures might be caused by multiple mechanisms. AQR has been linked with RNA splicing and R-loop suppression (*24*, *47*), and AQR was suggested to promote HR in response to different DSB-inducing agents (*29*). Collectively, these functional perturbations may jointly underlie genome instability in *AQR* hemizygous cells. We found helicases, including *AQR*, to be recurrently mutated in breast cancers, a cancer type with molecularly defined subtypes, each with distinct biology, prognosis, and response to therapy (*48*, *49*). We note that *TP53* mutations, frequently co-occurring with *AQR*, is frequent in Basal-like and HER2-enriched tumors, and it will be interesting to study subtype specificities in terms of recurrence, molecular patterns, and disease outcome in a future well-powered study. *AQR* is a common essential gene, and targeting such essential genes therapeutically can prove difficult due to high toxicity, although it is notable that the drugging of essential kinases such as WEE1, ATR, and CHK1 are being explored in multiple clinical trials (*50*, *51*). Thus, it is interesting to speculate that cells with hemizygous loss of *AQR* could be more vulnerable to AQR-targeted therapies than normal diploid cells. More directly, our findings also support a beneficial effect of PARP inhibitor treatment of *AQR* haploinsufficient tumors. Together, we propose that subset of helicases are driving cancer development through a mechanism involving hemizygous loss and that the affected pathways present targetable cancer vulnerabilities. Functional-mechanistic experiments and larger cancer-focused analyses will be needed to explore and expand the role and implication of hemizygous drivers in cancer development and treatment.

## MATERIALS AND METHODS

### Cell culture

The human osteosarcoma cell line, U-2 OS, was cultured in Dulbecco’s modified Eagle’s medium (DMEM) supplemented with 10% fetal bovine serum (FBS; HyClone, HYCLSV30160.03) and 1% penicillin/streptomycin (GIBCO, 15140-130). The human breast epithelial cell line MCF10A was cultured in DMEM F-12 (GIBCO, 31330095) supplemented with 5% horse serum (GIBCO, 26050088), 1% penicillin/streptomycin (GIBCO,), insulin (10 μg/ml; Sigma-Aldrich, I1882-100MG), hydrocortisone (0.5 μg/ml; Sigma-Aldrich, H0888-1G), epidermal growth factor (20 ng/ml; PeproTech, AF-100-15), and cholera toxin (100 ng/ml; Sigma-Aldrich/Merck, C8052-5MG).

### CRISPR and siRNA screens

Cherry-pick libraries targeting the 96-selected helicase and nuclease genes were purchased from Sigma-Aldrich (siRNA) and Dharmacon (crRNA) containing three siRNAs or guide RNAs to target each gene, respectively. The crRNA library contained 91 genes due to targeting issues with five genes (*BTBD12*, *CHD3*, *MTMR15*, *PRIC285*, and *RNASEN*). Location in the 384-well plate of crRNAs was randomized using an ECHO liquid dispenser to avoid location bias. The screens were performed using a Hamilton STARlet liquid dispenser. For the CRISPR screen, Cas9 expression was induced 24 hours before transfection using Doxycycline (Dox; 1 μg/ml). MCF10A iCas9 p53KO or U-2 OS cells were reversely transfected with 10 nM silencer select siRNA or 25 nM crRNA:tracrRNA complex. Seventy-two hours posttransfection, cells were fixed in 4% formaldehyde (VWR) for 15 min, followed by four washes with phosphate-buffered saline (PBS). Afterward, cells were permeabilized in 0.25% Triton X-100 for 10 min and washed four times with PBS before blocking in 3% bovine serum albumin (BSA) for 1 hour at room temperature. Incubation with the primary antibody targeting γH2AX (Merck Millipore, 05-636, 1:1000) was performed overnight. The following day, four PBS washes were performed followed by 1-hour incubation at room temperature with the anti-mouse Alexa Fluor 488 antibody (Invitrogen, A-21202), four PBS washes, 30-min incubation with DAPI (1 μg/ml), and five final washes with PBS. Sixteen imaging fields were acquired with an IN Cell Analyzer 2200 microscope and a 20x objective (GE Healthcare) to obtain ~2000 cells per well for control samples for the analysis with the IN Cell Analyzer Workstation (Cytiva) software. Nuclei were segmented on the basis of DAPI staining using the tophat segmentation method, and the mean γH2AX intensity was measured within the nuclei.

The data analysis was performed as described in (*52*), based on the percentage of γH2AX-positive cells per well and biological triplicates. In addition, the strictly standardized mean difference (SSMD) was calculated per gene, based on the normalized levels of γH2AX-positive cells to the negative control (*53*).

### siRNA transfections

Cells were seeded the day before transfection with 50 nM siRNA using Lipofectamine RNAiMAX (Thermo Fisher Scientific, 13778500). The media were changed or cells were reseeded 5 hours posttransfections. The MISSION siRNA Universal Negative Control #1 (siUNC, Sigma-Aldrich, SIC001-10NMOL) was used as a negative control. All siRNA sequences can be found in table S6.

### Generation of p53KO, heterozygous AQR, and BRCA2 3308 mutation cell lines

MCF10A iCas9 cells were transfected with an all-in-one pSpCas9(BB)-2A-GFP plasmid (PX458, Addgene 48138), containing a CRISPR guide sequence targeting TP53 exon 2 (TCGACGCTAGGATCTGACTG). Cells were sorted for medium Cas9-GFP expression using a FACS Aria III (BD) and treated with 10 μM Nutlin3a (Tocris Bioscience, 6075) to enrich for clones with p53 knockout. Single-cell clones were screened for p53 knockout by Indel Detection by Amplicon Analysis (IDAA) and validated by sequencing (*54*). Twelve clones, each with a homozygous loss of p53, were pooled and used for further experiments.

To generate MCF10A iCas9 p53KO cells heterozygous for AQR, cells were seeded and Cas9 expression was induced by treatment with Dox (1 μg/ml). The following day, cells were transfected with 15 nM crRNA (UAGGCCUUGCUAGAUACCUC):tracrRNA complex using Lipofectamine RNAiMAX. Two days posttransfection, cells were single-cell sorted into 96-well plates using a FACS Aria III (BD). Indel formation was analyzed by IDAA, and cells with a frameshift indel as well as a WT clone as an internal control were selected for further testing. The initial passage (p1) was used for phenotypic evaluation and sequencing and was cultured for 30 passages (p31) over 15 weeks and treated for 16 hours with aphidicolin (0.5 μg/ml) once a week.

To generate BRCA2 Y3308* mutation in MCF10A iCas9 cells, cells were seeded and Cas9 expression was induced by treatment with Dox (1 μg/ml) 24 hours in advance. The following day, cells were transfected with 20 nM crRNA (UAGGCCUUGCUAGAUACCUC):tracrRNA complex using Lipofectamine RNAiMAX together with 10 nM single-stranded nucleotide donor carrying the mutation. Two days posttransfection, cells were single-cell sorted into 96-well plates using a FACS Aria III (BD). The knock-in formation was analyzed by restriction enzyme digestion, with a single site introduced by the donor, as well as a WT clone as an internal control was selected for further testing.

### Library preparation for WGS

AQR WT and heterozygous clones from p1 and p31 were single-cell sorted into 96-well plates using a BD flow cytometer. Single-cell clones were grown, and cell pellets with 500,000 cells were frozen for sequencing. High-molecular-weight DNA was extracted using the Gentra Purigene Cell kit (Qiagen, 158767) following the manufacturer’s instructions for culturing cells. Briefly, cells were lysate using 300 μl of Cell Lysis Solution, followed by RNase treatment. After 5-min incubation at 37°C, 100 μl of Protein Precipitation Solution was added to the lysate and remove proteins. After centrifugation for 1 min at 15,000*g*, precipitated proteins form a tight pellet. The supernatant was transferred into a clean 1.5-ml microcentrifuge tube with 300 μl of isopropanol. After mixing and spin down, DNA was visible as a white pellet. Final wash with 300 μl of 70% ethanol was done to remove remain impurities. DNA was resuspended in 100 μl of DNA Hydration Solution and incubated at 65°C for 1 hour to dissolve it. DNA concentration was measured by the Qubit dsDNA BR Assay Kit (Thermo Fisher Scientific, Q32850), purity by Denovix spectrophotometer (DS-11Fx), and DNA integrity by electrophoresis in 0.8% agarose gel.

We performed shallow WGS on 113 cell line clones, using 400 ng of genomic DNA to prepare polymerase chain reaction (PCR) free libraries using the Illumina DNA PCR-free kit (20041794). This strategy provides several benefits for accurate sequencing, i.e., eliminate PCR duplicates and polymerase errors. It combines tagmentation on beads with extension using IDT for Illumina DNA Unique Dual Indexes (Illumina DNA PCR-Free Library Prep, Reference Guide 1000000086922 v01). Final libraries were quantified by the Qubit ssDNA Assay Kit (Thermo Fisher Scientific, Q10212), and size distribution was assessed by the Bioanalyzer High Sensitivity DNA Kit (Agilent, 5067-4626). Libraries were combined at a 2 nM concentration pool and paired-end sequenced using NextSeq 500/550 High Output Kit v2.5 (150 cycles; Illumina, 20024907).

As a validation, deep WGS was done on six randomly selected samples. Libraries were prepared using 200 ng of genomic DNA following the NEBNext Ultra II FS DNA Library Prep Kit for Illumina (New England Biolabs, E7805S). Briefly, DNA was enzymatically fragmented for 12 min at 37°C to get 450-bp fragments on average. After size selection, end repair, dA tailing, and UDI Adaptor Ligation, final libraries were amplified by five PCR cycles and quantified by the Qubit dsDNA High Sensitivity assay Kit (Thermo Fisher Scientific, Q10212). Size distribution was assessed by the Bioanalyzer High Sensitivity DNA Kit (Agilent, 5067-4626). Last, libraries were combined at a 2 nM concentration pool and paired-end sequenced using 300 cycles on the NovaSeq 6000 S2 Reagent Kit v1.5 (300 cycles; Illumina, 20028314). Variant calling was performed as described in (*55*).

### Generation of AQR-GFP inducible MCF10A cells

For lentivirus production, human embryonic kidney (HEK) 293T cells were transfected with 3 μg of pLVX, 1 μg of VSV-G (Clontech), and 1 μg of PAX2 (Clontech) plasmids using JetPEI (Polyplus, 101-10 N) according to the manufacturer’s protocol. Five hours posttransfection, the transfection media were aspirated and cells were cultured in 10 ml of MCF10A media. Two days after plasmid transfection, lentiviral particles were collected by centrifugation of the supernatant at 300*g* for 5 min. For the transduction, 7 ml of the supernatant was mixed with 3 ml of MCF10A culture media, and polybrene (Sigma-Aldrich, H9268-5G) was added at a final concentration of 10 μg/ml. MCF10A cells were transduced on 2 consecutive days, and cells were selected with puromycin (3 μg/ml). In addition, AQR-GFP expression was induced with Dox (1 μg/ml) for 48 hours, and cells were sorted for GFP expression using a FACS Aria III (BD). Different GFP expression levels were tested by Western blotting to match with endogenous AQR expression. The pLVX plasmid containing only the GFP sequence was used to generate GFP-inducible expressing cells.

### R-loop testing by RNase H1 overexpression and DRB treatment

U-2 OS cells were transfected with siRNAs as described above followed by reseeding and, 48 hours later, plasmid transfection of either pEGFP or pEGFP-RNase H1 (M27) plasmids (*56*) using GenJet (SignaGen Laboratories, SL100489-OS) according to the manufacturer’s protocol. Twenty-four hours post–plasmid transfection, cells were harvested and prepared for immunofluorescence or immunoblotting as described below. γH2AX mean intensity was measured for GFP-positive cells only.

To assess the effect of transcription inhibition on genome instability, MCF10A iCas9 p53KO cells were transfected with siRNA and, after 66 hours, treated with 150 μM DRB (5,6-dichloro-1-β-d-ribofuranosylbenzimidazole; Sigma-Aldrich, D1916-10MG) for 6 hours. Samples were prepared for immunofluorescence and immunoblotting.

### Homologous recombination assay

U-2 OS cells transfected with the pDR-GFP plasmid (Addgene, 26475) using GenJet (SignaGen Laboratories, SL100489-OS) according to the manufacturer’s protocol were selected with puromycin (5 μg/ml). To investigate HR efficiency, cells were seeded in a 6-well plate and, the next day, transfected with siRNA followed by a media change 5 hours posttransfection. Twenty-four hours post–siRNA transfection, cells were transfected with the I-Sce1 plasmid using GenJet according to the manufacturer’s protocol. Two days later, cells were trypsinized and fixed in 1% formaldehyde for 15 min, followed by PBS wash and permeabilization in 60% EtOH for 1 hour on ice. Afterward, cells were washed and stained with propidium iodide (PI) in the presence of RNase A for 30 min at 37°C. Samples were analyzed on a FACSCalibur using the Cellquest Pro (BD Biosciences) and FlowJo software. The HR efficiency was calculated for 20,000 cells per sample based on the percentage of GFP-positive cells in S and G_2_ phases of the cell cycle and normalized to siUNC-transfected cells. The MCF10A *BRCA2* homozygous Y3308* mutant was classified as pathogenic by ClinVar (NCBI; VCV000052916.29, https://ncbi.nlm.nih.gov/clinvar/variation/VCV000052916.29 (accessed 3 June 2023).

### PARP inhibitor sensitivity assay

For each sample, 1000 cells were seeded in a well of a 6-well plate, using the AQR WT or heterozygous clones and a BRCA2 3308* pathogenic variant in MCF10A iCas9 cells as a positive control. Three technical replicates were performed to account for seeding differences. Two days after seeding cells, cells were treated with the indicated doses of talazoparib or dimethyl sulfoxide (DMSO) for 4 days. Afterward, pretreated cells were counted and reseeded at a density of 200 cells per well. After another 2 days, cells were treated again with the indicated doses of talazoparib for another 6 days. Cells were fixed in 100% methanol for 10 min, followed by staining with MeOH/Crystal Violet for another 10 min. Colonies were manually counted, and numbers were normalized to DMSO-treated cells.

### Immunoblotting

Radioimmunoprecipitation assay (RIPA) buffer (Sigma-Aldrich, R0278) supplemented with 1% (v/v) aprotinin, leupeptin (5 μg/ml), 1x cOmplete EDTA-free protease inhibitor cocktail (Sigma-Aldrich, 5056489001), 5 mM NaF, 20 mM β-glycerophosphate, and 0.2 mM Na_3_VO_4_ was used to lyse cells on ice. A 102C CE Converter, Branson, was used to sonicate all samples followed by centrifugation at 20,000*g* for 10 min. The protein concentration was measured by Bradford assay. SDS–polyacrylamide gel electrophoresis (PAGE) was used to resolve proteins, and proteins were transferred to a nitrocellulose membrane. Membranes were blocked in 5% milk in Phosphate-Buffered Saline with Tween 20 (PBS-T) for 1 hour at room temperature followed by incubation with the primary antibody overnight. Before incubation with anti-rabbit or anti-mouse secondary horseradish peroxidase (HRP)–conjugated antibodies (Vector Laboratories, PI-1000 and PI-2000), and afterward, membranes were washed for 30 min in PBS-T. Information about the antibody manufacturer, catalog number, and used dilution can be found in table S6.

### Immunofluorescence

If applicable, cells were pulsed with 10 μM EdU (Thermo Fisher Scientific, A10044) for 30 min before fixation with 4% formaldehyde for 12 min. After three PBS washes, cells were permeabilized in 0.25% Triton X-100 in PBS for 10 min and again washed three times with PBS. Cells were blocked in 3% BSA in DMEM containing 10% FBS for 20 min at room temperature. To visualize EdU incorporation, the Click-IT cell reaction buffer kit was used according to the manufacturer’s instructions (Thermo Fisher Scientific, C10269) together with Alexa Fluor 647 Azide (Thermo Fisher Scientific, A10277). Primary and secondary antibodies were prepared in blocking solution and incubated for 1 hour at room temperature. To stain nuclei, DAPI (Sigma-Aldrich, D9542-5MG) was incubated together with the secondary antibody at a final concentration of 0.1 μg/ml. Imaging was performed using an Olympus ScanR workstation, and the respective software was used for the analysis.

### Immunofluorescence statistics

All immunofluorescence-based analyses of γH2AX mean intensity were performed in three biological replicates, each containing technical triplicates. All graphs represent one biological replicate due to a large amount of data. Each technical replicate contributes equally to the panels represented in the figures, e.g., *n* = 450 means that 150 nuclei were randomly selected from one technical replicate. Nonparametric statistical analyses were performed on a rank that was assigned to each nuclei based on its γH2AX mean intensity. A linear mixed model was fitted for the ranks, and the technical replicates were used as a variance component to account for biological heterogeneity within an experiment. Multiple comparisons were performed using the lsmeans and the contrast function of the lsmeans package in R (https://rdocumentation.org/packages/lsmeans/versions/2.27-2/topics/lsmeans). *P* values were adjusted using the Holm method. Micronuclei analysis was performed as described above but included data from biological as well as technical replicates. The DR-GFP assay was analyzed using a Mann-Whitney *U* test to compare siUNC to either siAQR- or siBRCA2-transfected cells. PARP inhibitor sensitivity was analyzed using a two-way analysis of variance (ANOVA) test for repeated measures and Dunnet’s multiple comparisons. *P* values are reported with the following significance codes: *P < 0.05, **P < 0.001, ***P < 0.001, and ****P < 0.0001.

### Site-directed mutagenesis

siRNA-resistant AQR plasmids for lentivirus production were generated using site-directed mutagenesis followed by InFusion. As a template, the AQR-GFP plasmid (RG220742) was used for the mutagenesis with KOD polymerase (Merck Millipore, 71086-3), used according to the manufacturer’s protocol. The siRNA-resistant AQR sequence was cloned into the pLVX-TetOne vector (Clontech Laboratories), containing an N-terminal GFP, using the InFusion HD cloning kit (Clontech Laboratories, 639691) according to the manufacturer’s protocol. Primer sequences can be found in table S6.

### Somatic variant analysis of pan-cancer data

PCAWG data analysis is based on 2581 WGS profiled tumor samples from the PCAWG consortium (*12*) with 31 histology types (pan-cancer) and 212 unique breast cancer patient samples. Analysis of genomic aberrations was conducted using the official release set of somatic point mutation, copy number alterations, and SVs (*57*). Every gene was annotated as mutant if one or several of the following requirements were satisfied: (i) nonsynonymous somatic or germline SNV in the gene, (ii) somatic copy number loss overlapping at least 50% of the gene body, and/or (iii) SV predicted to disrupt the open reading frame of the gene in question. A series of analyses was performed for SV breakpoint recurrence. SV breakpoint was annotated with length of nearby sequence homology, binned by 0 to 3, 4 to 11, and 12 to 1000 nt. We computed the proportion of each group, by normalizing to the total number of SVs in each sample. Analysis of deletion and tandem duplication (TD) size distributions was performed, by counting the number of deletions and the number duplications in binned sizes of 0 to 1 kb, 1 to 10 kb, 10 to 100 kb, 100 to 1000 kb, and >1 Mb and normalized to the total number of TDs. SBS signature exposure was computed by normalizing the number of inferred SNVs attributed to that SBS to the total number of SNVs for each tumor sample.

Genome instability from TCGA and PCAWG was computed as the percentage of the aberrant genome using the weighted genome instability index (WGII) in which the portion of the aberrant genome is calculated by chromosome, and the mean proportion of the aberrant genome is calculated across the chromosomes. This approach prevents the inflation of the score caused by aberrations in larger chromosomes.

We used the publicly available PCAWG segmentation release data, including ploidy and purity estimates. We defined the aberrant segments when the copy number estimated for the segment was different from the estimated ploidy and used the cumulative aberrant segments size to estimate the proportion of aberration relative to the chromosome size.

For TCGA data, we used publicly available segmented data from single-nucleotide polymorphism (SNP) arrays (cBioPortal), we computed the genome mean log*R* value from the segment data and estimated aberrant segments those outside the log*R* range of −0.2 and 0.2. We then calculated the WGII score in a similar manner than the PCAWG cohort.

### Mutation enrichment per enzyme family

We collected publicly available mutation data from the TCGA (https://portal.gdc.cancer.gov, 8 June 2017), comprising Bladder carcinoma (BLCA 99 patients), Breast carcinoma (BRCA 892 patients), Colorectal adenocarcinoma (COAD 233 patients), Esophageal adenocarcinoma (ESCA 141 patients), Head and neck carcinoma (HNSC 384 patients), Kidney clear cell carcinoma (KIRC 417 patients), Kidney renal papillary cell carcinoma (KIRP 291 patients), Low-grade glioma (LGG 515 patients), Liver Hepatocellular Carcinoma (LIHC 377 patients), Lung adenocarcinoma (LUAD 405 patients), Lung squamous cell carcinoma (LUSC 178 patients), Ovarian carcinoma (OV 316 patients), Pancreatic adenocarcinoma (PAAD 185 patients), Prostate adenocarcinoma (PRAD 499 patients), Rectum adenocarcinoma (READ 167 patients), Melanoma (SKCM 118 patients), Thyroid Cancer (THCA 503 patients), and Thymoma (THYM 124 patients).

We identified the genes belonging to the seven enzyme classes (namely, oxidoreductases, transferases, hydrolases, lyases, isomerases, ligases, and translocases) from UniProt and 12 enzyme families (namely, glycosidase, G protein, kinase, lipase, methyltransferase, oxidoreductase, oxygenase, phosphatase, protease, transaminase, helicase, and nuclease) as classified in pantherdb (http://www.pantherdb.org). Sixty-six of 69 oxygenase genes were redundant with oxidoreductases, and we therefore excluded this family from further analyses due to the low number of unique genes. We computed the number of missense and nonsense mutation, splice site, and frameshift deletions for each gene using the TCGA cohort. As an additional criterion, we considered only genes that were mutated in at least two different patients in the same cancer type.

We used a permutation approach to compute the probability of observing a given number of mutated genes by randomly sampling gene lists matched in number and within ±5% of total genomic coverage and repeating the process 10,000 times, for each enzyme class, family, and for each cancer type. We calculated the empirical *P* value given by the time an equal or higher number of genes were found mutated with respect to the observed number of mutations, divided by the number of iterations.

For each cancer type enzyme class/family combination, we also computed the SMD, subtracting the mean of the mutated genes in the permutations to the number of observed mutated genes and dividing by the SD of the permutations.

We computed a single *P* value for each enzyme class/family using the Fisher combination test on the empirical *P* value for each cancer type.

To obtain an aggregate effect size estimate for each enzyme class/family, we calculated the mean of the SMD for all cancer types. We used the information of the 5th and 95th percentiles, as a measure for cancer-type specific effect sizes.

Enzyme class enrichment analysis in COSMIC CGC catalog of known driver genes (version 95) was performed by comparing the number CGC genes in each enzyme family to the expected proportion using a Fisher’s exact test (see also table S2).

### Permutation-based significance assessment of cancer mutation data

To have a background model to compare various analyses on the PCAWG cohort, we applied the curveball algorithm (*58*) to the binary matrix of the mutational data, in which columns represent genes and the rows represent patients.

The curveball algorithm constrains the randomization to scenarios where the cumulative sums of each column and row are always constant, providing a background model closer to a real scenario compared to an unbounded or partially bounded shuffling methodology.

We applied the method to obtain 10,000 permuted versions of the mutation binary matrix.

### Estimation of the gene expression effect on mutation rate

To investigate whether gene expression levels influenced mutation state, we developed a comprehensive pan-cancer analysis framework. For each gene, we calculated a normalized mutation rate by dividing the number of deleterious mutations (including missense, nonsense, frameshift, and in-frame variants) by gene length (in kilobases). We then used linear regression models to test whether gene expression levels predict mutation rates while controlling for gene length and accounting for zero inflation in the mutation rate distribution. The analysis was performed separately for different functional gene classes across the 18 TCGA cancer types selected in this study, with a minimum requirement of three mutation events per gene to ensure robust statistical inference. Statistical significance was assessed using Benjamini-Hochberg adjusted *P* values within each gene class. Table S1 presents a comprehensive pan-cancer analysis of how gene expression levels influence mutation rates across different functional gene classes. For each cancer type and gene class combination, we collected a summary of the linear model results, indicating among other information, the mean estimated effect size (average change in mutation rate per unit increase in expression), and the percentage of tested genes showing significant association.

A positive effect size indicates that higher expression is associated with increased mutation rate, whereas negative values suggest that higher expression correlates with lower mutation rates.
