# Supplementary material for: Hemizygous loss of helicases promotes genomic instability and cancer development
Source: Sci Adv. 2026 Feb 20;12(8):eadv4540. doi: 10.1126/sciadv.adv4540 (PMC12922743; doi:10.1126/sciadv.adv4540)
Supplement: Supplementary file 1 — Figs. S1 to S10 Legends for tables S1 to S6 [file sciadv.adv4540_sm.pdf]

Science Advances
AAAS

Supplementary Materials for

Hemizygous loss of helicases promotes genomic instability and cancer development

Karolin Voßgröne *et al.*

Corresponding author: Claus S. Sørensen, claus.storgaard@bric.ku.dk;
Joachim Weischenfeldt, joachim.weischenfeldt@bric.ku.dk

Sci. Adv. **12**, eadv4540 (2026)
DOI: 10.1126/sciadv.adv4540

The PDF file includes:

Figs. S1 to S10
Legends for tables S1 to S6

Other Supplementary Material for this manuscript includes the following:

Tables S1 to S6

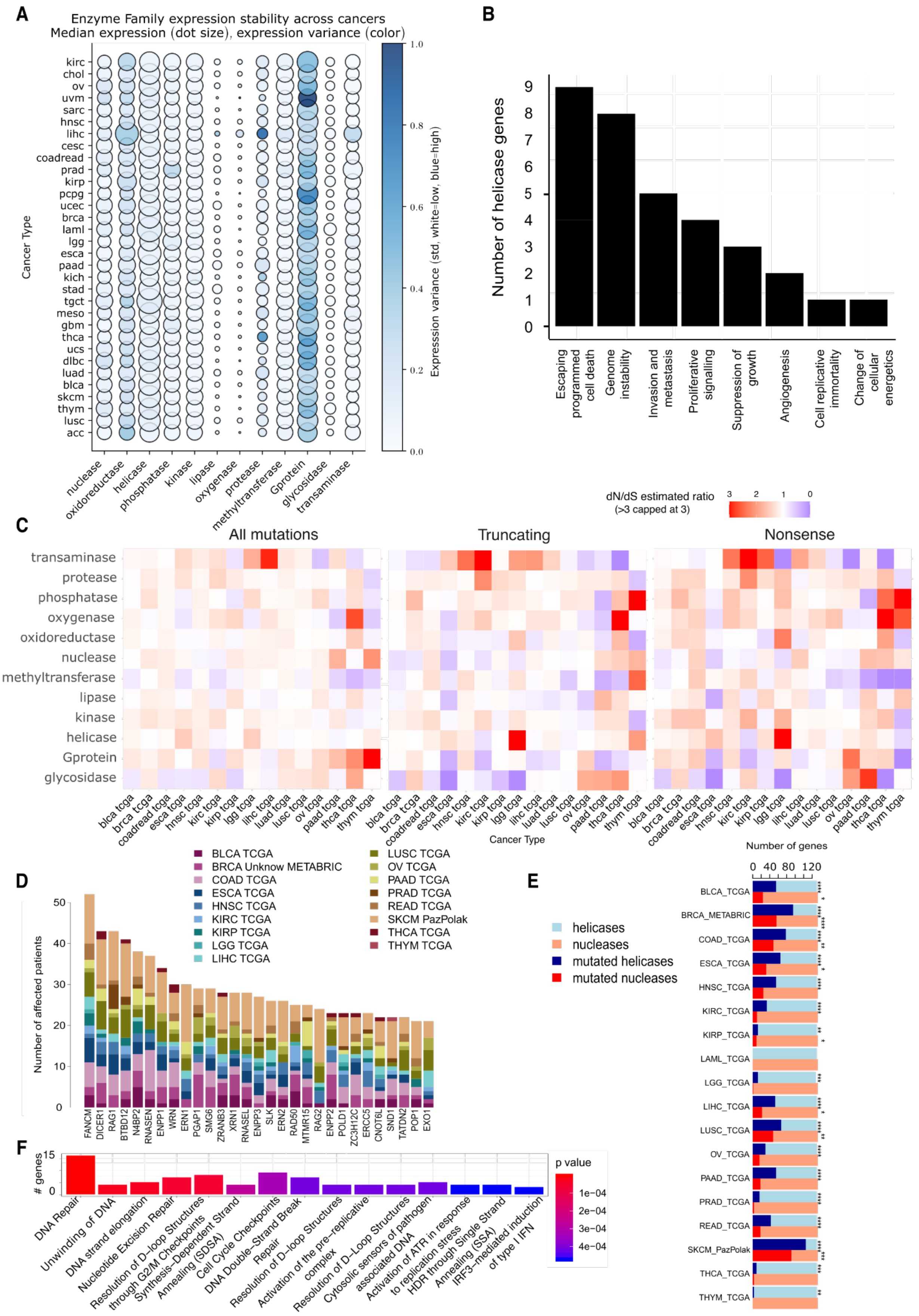
A
Enzyme Family expression stability across cancers
Median expression (dot size), expression variance (color)
Cancer Type
kirc
chol
ov
uvm
sarc
hnsc
lihc
cesc
coadread
prad
kirp
pcpg
ucec
brca
laml
lgg
esca
paad
kich
stad
tgct
meso
gbm
thca
ucs
dlbc
luad
blca
skcm
thym
lusc
acc
nuclease
oxidoreductase
helicase
phosphatase
kinase
lipase
oxygenase
protease
methyltransferase
Gprotein
glycosidase
transaminase
Expresssion variance (std, white=low, blue=high)
1.0
0.8
0.6
0.4
0.2
0.0
B
Number of helicase genes
Escaping programmed cell death
Genome instability
Invasion and metastasis
Proliferative signalling
Suppression of growth
Angiogenesis
Cell replicative immortality
Change of cellular energetics
C
dN/dS estimated ratio
(>3 capped at 3)
All mutations
Truncating
Nonsense
transaminase
protease
phosphatase
oxygenase
oxidoreductase
nuclease
methyltransferase
lipase
kinase
helicase
Gprotein
glycosidase
blca tcga
brca tcga
coadread tcga
esca tcga
hnsc tcga
kirc tcga
kirp tcga
lgg tcga
lihc tcga
luad tcga
lusc tcga
ov tcga
paad tcga
thca tcga
thym tcga
Cancer Type
D
Number of affected patients
BLCA TCGA
BRCA Unknow METABRIC
COAD TCGA
ESCA TCGA
HNSC TCGA
KIRC TCGA
KIRP TCGA
LGG TCGA
LIHC TCGA
LUSC TCGA
OV TCGA
PAAD TCGA
PRAD TCGA
READ TCGA
SKCM PazPolak
THCA TCGA
THYM TCGA
FANCM
DICER1
RAG1
BTBD12
N4BP2
RNASEN
ENPP1
WRN
ERN1
PGAP1
SMG6
ZRANB3
XRN1
RNASEL
ENPP3
SLK
ERN2
RAD50
MTMR15
RAG2
ENPP2
POLD1
ZC3H12C
ERCC5
CNOT6L
SND1
TATDN2
POP1
EXO1
E
Number of genes
helicases
nucleases
mutated helicases
mutated nucleases
BLCA_TCGA
BRCA_METABRIC
COAD_TCGA
ESCA_TCGA
HNSC_TCGA
KIRC_TCGA
KIRP_TCGA
LAML_TCGA
LGG_TCGA
LIHC_TCGA
LUSC_TCGA
OV_TCGA
PAAD_TCGA
PRAD_TCGA
READ_TCGA
SKCM_PazPolak
THCA_TCGA
THYM_TCGA
F
genes
p value
DNA Repair
Unwinding of DNA
DNA strand elongation
Nucleotide Excision Repair
Resolution of D-loop Structures through Synthesis-Dependent Strand Annealing (SDSA)
G2/M Checkpoints
Cell Cycle Checkpoints
DNA Double-Strand Break Repair
Resolution of D-loop Structures
Activation of the pre-replicative complex
Resolution of D-Loop Structures
Cytosolic sensors of pathogen associated DNA
Activation of ATR in response to replication stress
HDR through Single Strand Annealing (SSA)
IRF3-mediated induction of type I IFN

Fig. S1. Cancer genomic analyses by enzyme families across TCGA cohorts.
A) Expression summary by enzyme family and cancer type (TCGA). Color intensity indicates the standard deviation and dot size indicate the median expression (TPM).
B) Hallmarks of Cancer associated with helicases in CGC.
C) Most likely estimates for the dN/dS ratios in all, nonsense and truncating mutations across the enzyme families in different cancer types across the TCGA cohort.
D) Occurrence of somatic variants in nucleases: the 30 most frequently mutated nuclease genes ordered by occurrence. Each column corresponds to a gene, the colours in each column identify different tumour types, the height of a column corresponds to the number of patients in the 17 tumour-type studies (legend) carrying a mutation in the corresponding gene. See Supplementary Table 1 for the full list.
E) Number of helicase and nuclease genes mutated in 18 different tumour-type studies from TCGA and METABRIC. Asterisks denote significance level (*, $P<0.05$; **, $P<0.01$; *** $P<0.001$, **** $P<0.0001$ Fisher's exact test)
F) Pathway analysis of mutated helicase and nuclease genes in breast cancer (BRCA METABRIC study). Pathways sorted by level of p-value significance (y-axis). X-axis represents the number of genes in each pathway and the colour scale represents the adjusted p-value significance level.

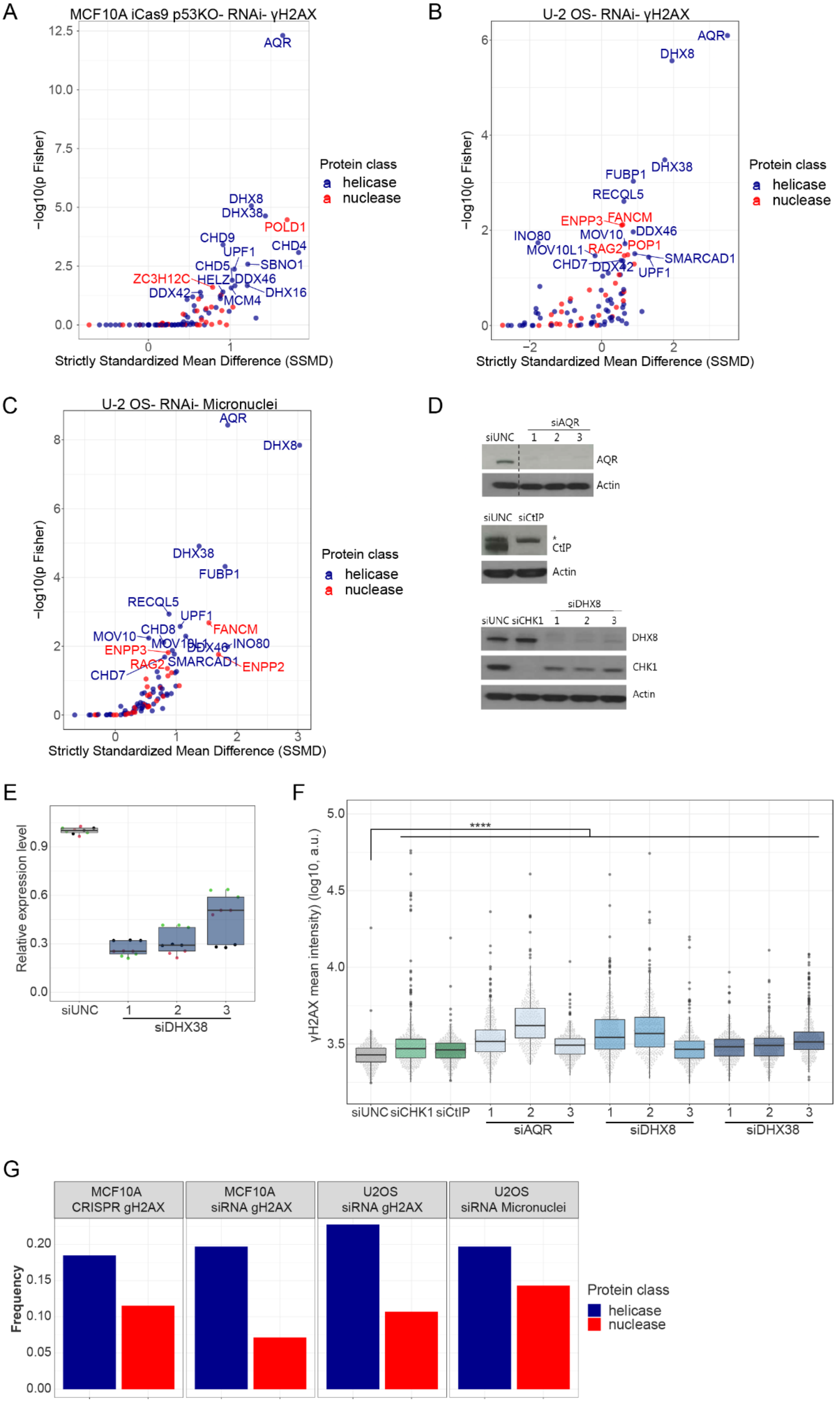

A
MCF10A iCas9 p53KO- RNAi- γH2AX
AQR
DHX8
DHX38
POLD1
CHD9
CHD4
UPF1
CHD5
SBNO1
ZC3H12C
HELZ
DDX46
DDX42
DHX16
MCM4
−log10(p Fisher)
Strictly Standardized Mean Difference (SSMD)
Protein class
helicase
nuclease
B
U-2 OS- RNAi- γH2AX
AQR
DHX8
DHX38
FUBP1
RECQL5
ENPP3
FANCM
INO80
MOV10
DDX46
MOV10L1
RAG2
POP1
SMARCAD1
CHD7
DDX42
UPF1
−log10(p Fisher)
Strictly Standardized Mean Difference (SSMD)
Protein class
helicase
nuclease
C
U-2 OS- RNAi- Micronuclei
AQR
DHX8
DHX38
FUBP1
RECQL5
UPF1
CHD8
FANCM
MOV10
ENPP3
MOV10L1
DDX46
INO80
RAG2
SMARCAD1
ENPP2
CHD7
−log10(p Fisher)
Strictly Standardized Mean Difference (SSMD)
Protein class
helicase
nuclease
D
siUNC
siAQR
1
2
3
AQR
Actin
siUNC
siCtIP
*
CtIP
Actin
siUNC
siCHK1
siDHX8
1
2
3
DHX8
CHK1
Actin
E
Relative expression level
siUNC
1
2
3
siDHX38
F
γH2AX mean intensity) (log10, a.u.)
****
siUNC
siCHK1
siCtIP
1
2
3
siAQR
1
2
3
siDHX8
1
2
3
siDHX38
G
MCF10A CRISPR gH2AX
MCF10A siRNA gH2AX
U2OS siRNA gH2AX
U2OS siRNA Micronuclei
Frequency
Protein class
helicase
nuclease

Fig. S2. Supporting evidence for cancer-associated genome maintenance helicases and nucleases

A) MCF10A iCas9 p53KO siRNA screen results, performed as shown in Figure 2A. On the x-axis the effect size is plotted as the strictly standardised mean difference (SSMD) in the frequency of γH2AX positive cells. The y-axis corresponds to the Fisher combined p-value from three biological replicates. Coloured by enzyme family and annotated dots represent significantly scoring genes (p Fisher <0.05). B) U-2 OS siRNA screen results, performed as shown in Figure 2A. On the x-axis the effect size is plotted as the strictly standardised mean difference (SSMD) in the frequency of γH2AX positive cells. The y-axis corresponds to the Fisher combined p-value from three biological replicates. Coloured by enzyme family and labelled dots represent significantly scoring genes (p Fisher <0.05).

C) U-2 OS siRNA screen results for micronuclei formation, performed as shown in Figure 2A. On the x-axis the effect size is plotted as the strictly standardised mean difference (SSMD) in the frequency of micronuclei-positive cells. The y-axis corresponds to the Fisher combined p-value from three biological replicates. Coloured by enzyme family and annotated dots represent significantly scoring genes (p Fisher <0.05).

D) Immunoblot of samples in E-F and Figure 2F, assessing depletion of AQR, CtIP, CHK1 and DHX8, Actin served as a loading control. The dotted line indicates a non-continuous WB and an unspecific band detected by the CtIP antibody is labelled with an asterisk.

E) qPCR analysis of DHX38 knockdown of samples shown in F and Figure 2F. Point colours indicate samples belonging to the same biological replicate.

F) siRNA screen validation of AQR, DHX8 and DHX38-depleted U-2 OS cells. Timing as described in Figure 2A, depicting the γH2AX mean intensity. Representation of one out of three biological replicates, per sample n=450, n=150 per technical replicate. **** $P<0.0001$.

G) Analysis of the frequency of either helicase or nuclease genes scoring in each screen, normalised to the total number of helicase or nuclease genes in the target library. **** $P<0.0001$, *** $P<0.001$, * $P<0.05$, ns= not significant.

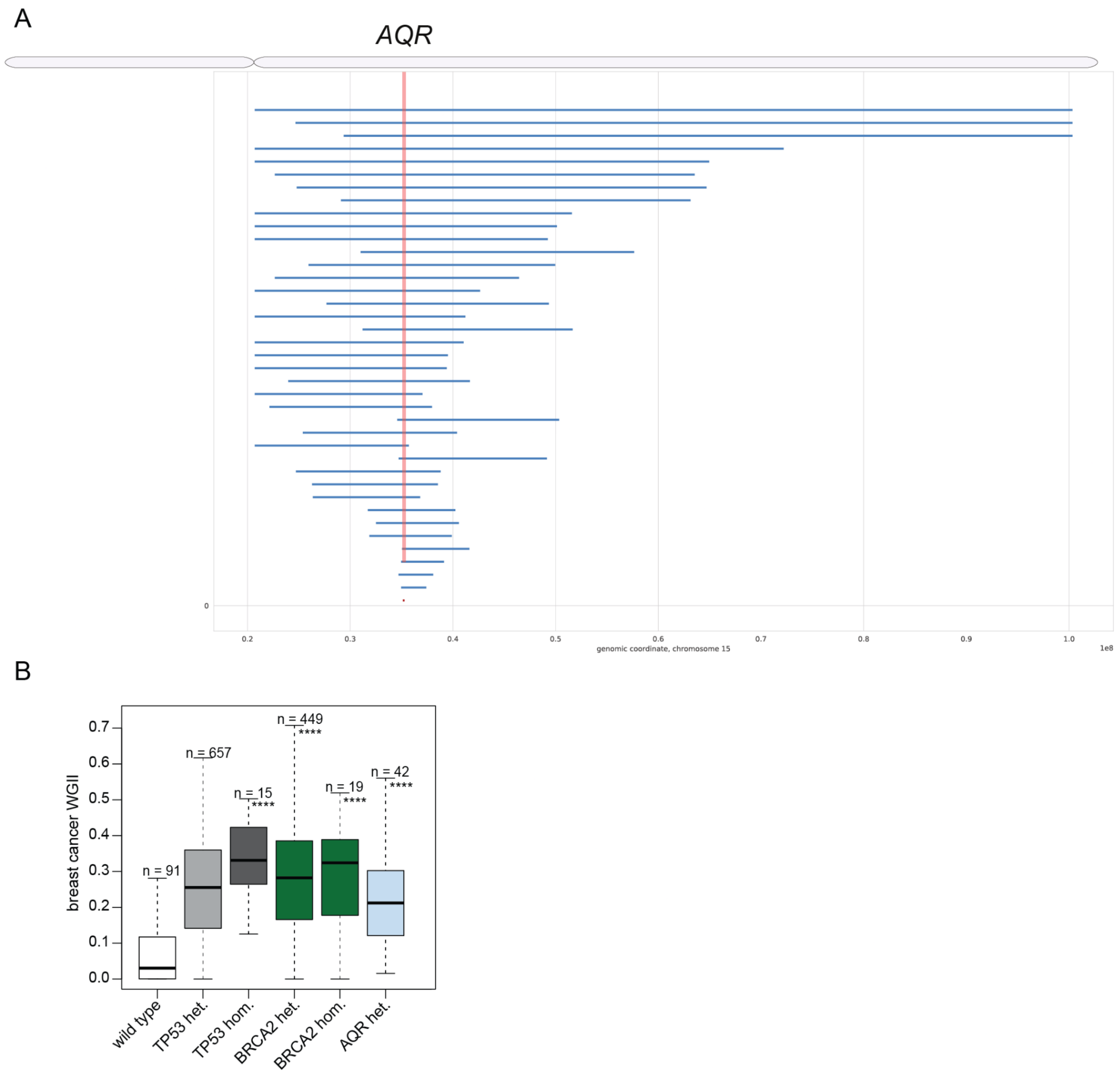

Fig. S3. Supporting evidence for hemizygous loss of AQR and associated genome instability

A) AQR tornado plot showing copy number alterations affecting AQR in breast cancer (PCAWG). Losses are displayed in blue and gains in red (none present).

B) Breast cancer analysis using TCGA breast cancer data of WGII for tumours with heterozygous or homozygous loss of *TP53*, *BRCA2* and *AQR*, considering samples without potential co-occurring alterations. No cancer samples had *AQR* homozygous loss (*P*=0.016, Fisher's exact test).

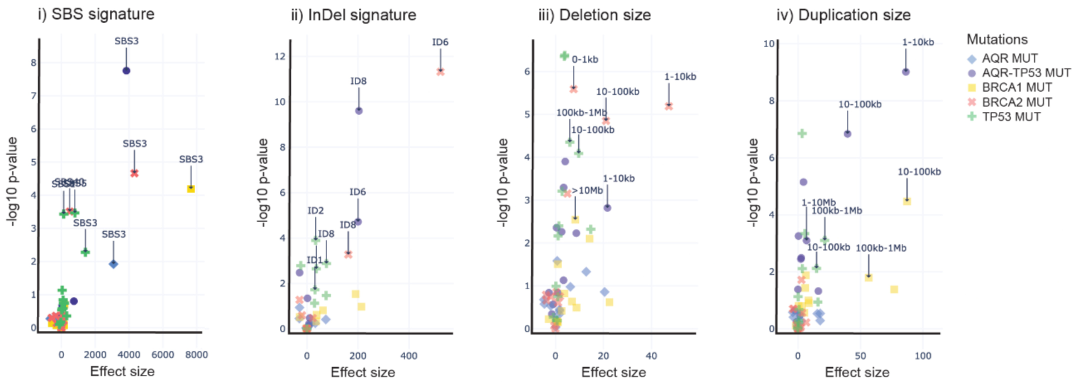

Fig. S4. Somatic signatures associated with AQR and TP53 loss

Linear model of mutational signatures regressed on the mutational status of hemizygous AQR loss, homozygous loss of TP53, of BRCA1 and of BRCA2 in PCAWG breast cancer samples. The figure shows the enrichment in mutational signature exposure of i) SBS signatures ii) InDel signatures, iii) Deletion sizes and iv) Duplication sizes. Coloured shapes represent the different mutual exclusive mutation types, with e.g. “AQR MUT” representing tumours with heterozygous loss of AQR but no homozygous loss of TP53, BRCA1 or BRCA2.

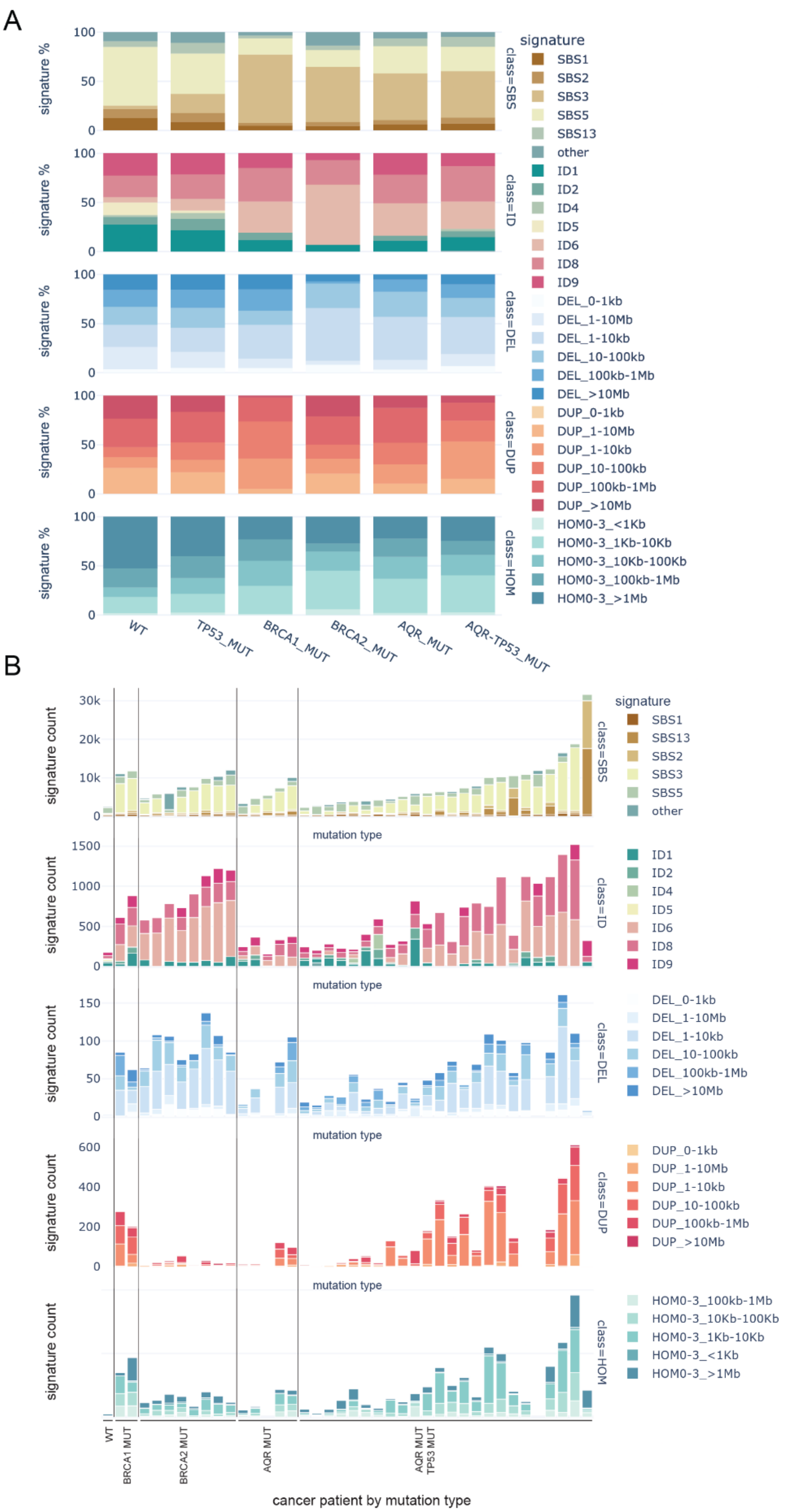

A
signature %
class=SBS
class=ID
class=DEL
class=DUP
class=HOM
100
50
0
signature
SBS1
SBS2
SBS3
SBS5
SBS13
other
ID1
ID2
ID4
ID5
ID6
ID8
ID9
DEL_0-1kb
DEL_1-10Mb
DEL_1-10kb
DEL_10-100kb
DEL_100kb-1Mb
DEL_>10Mb
DUP_0-1kb
DUP_1-10Mb
DUP_1-10kb
DUP_10-100kb
DUP_100kb-1Mb
DUP_>10Mb
HOM0-3_<1Kb
HOM0-3_1Kb-10Kb
HOM0-3_10Kb-100Kb
HOM0-3_100kb-1Mb
HOM0-3_>1Mb
WT
TP53_MUT
BRCA1_MUT
BRCA2_MUT
AQR_MUT
AQR-TP53_MUT
B
signature count
30k
20k
10k
0
1500
1000
500
150
100
600
400
200
mutation type
signature
SBS1
SBS13
SBS2
SBS3
SBS5
other
ID1
ID2
ID4
ID5
ID6
ID8
ID9
DEL_0-1kb
DEL_1-10Mb
DEL_1-10kb
DEL_10-100kb
DEL_100kb-1Mb
DEL_>10Mb
DUP_0-1kb
DUP_1-10Mb
DUP_1-10kb
DUP_10-100kb
DUP_100kb-1Mb
DUP_>10Mb
HOM0-3_100kb-1Mb
HOM0-3_10Kb-100Kb
HOM0-3_1Kb-10Kb
HOM0-3_<1Kb
HOM0-3_>1Mb
class=SBS
class=ID
class=DEL
class=DUP
class=HOM
WT
BRCA1 MUT
BRCA2 MUT
AQR MUT
AQR MUT TP53 MUT
cancer patient by mutation type

Fig. S5. Mutation signatures stratified by *AQR*/*TP53* status and tumour sample

A) Cumulative distribution of mutational signatures for breast cancer samples with *TP53* homozygous loss (TP53 MUT), *BRCA1* homozygous loss (BRCA1 MUT), *BRCA2* homozygous loss (BRCA2 MUT), *AQR* heterozygous loss (AQR MUT) or both *AQR* heterozygous loss and *TP53* homozygous loss (AQR-TP53 MUT) or none of these mutations (WT). Top row: SBS signatures, 2nd row: ID signature, 3rd row: copy number deletion size, 4th row: copy number duplication size, 5th row: short microhomologies at different copy number sizes.
Only signatures present in at least 2% of the samples are shown.
B) Same as A) but showing the numerical signature count for the different mutational profiles. Median of all the WT samples is shown for comparison.

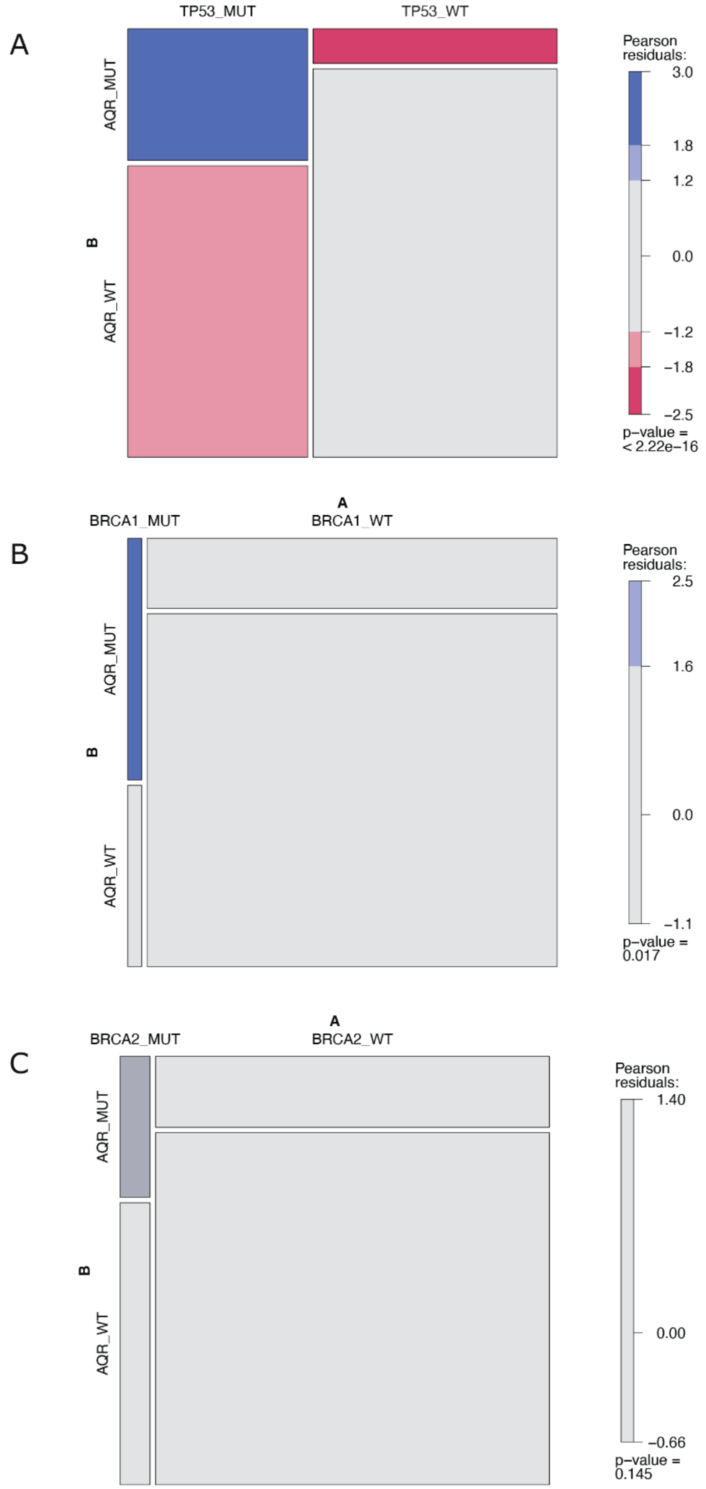

Fig. S6. Co-occurrence of *AQR* and *TP53* for breast cancer samples

A) Contingency table of breast cancer samples with heterozygous AQR loss (AQR MUT) and/or homozygous BRCA1 loss (BRCA1 MUT). The upper left square represents cancers with co-occurring mutations and the bottom right represents breast cancers without mutations in both

enzymes, the top right are cancers exclusively with *AQR* heterozygous loss and the bottom left is cancers exclusively with *TP53* homozygous loss.
B) Similar to A) but co-occurrence of heterozygous *AQR* loss (AQR MUT) and homozygous *BRCA1* loss (BRCA1 MUT) for breast cancer samples.
C) Similar to A) but co-occurrence of heterozygous *AQR* loss (AQR MUT) and homozygous *BRCA2* loss (BRCA2 MUT) for breast cancer samples.
p-values for Fisher's exact is shown at the bottom right, with a colour gradient top right side relative to the Pearson residuals of the co-occurrence. Blue represents co-occurrence and red represents mutual exclusivity.

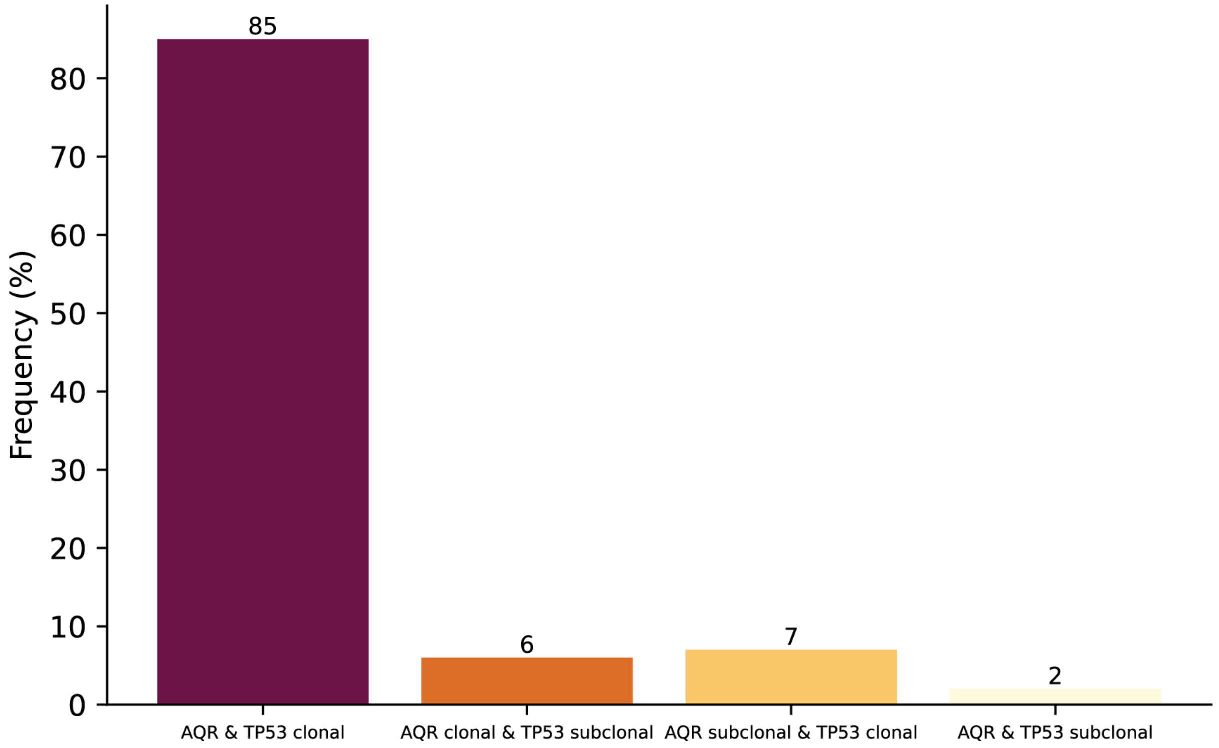

Fig. S7. Clonality of *AQR* and *TP53* mutated tumours

Frequency of breast cancers (n total=781) with clonal (ccf>0.95) or subclonal (ccf<=0.95) somatic copy number alterations separated by AQR and TP53 clonal (85%, purple), AQR clonal and TP53 subclonal (6%, orange), AQR subclonal and TP53 clonal (7%, dark yellow) and both AQR and TP53 subclonal (2%, pale yellow).

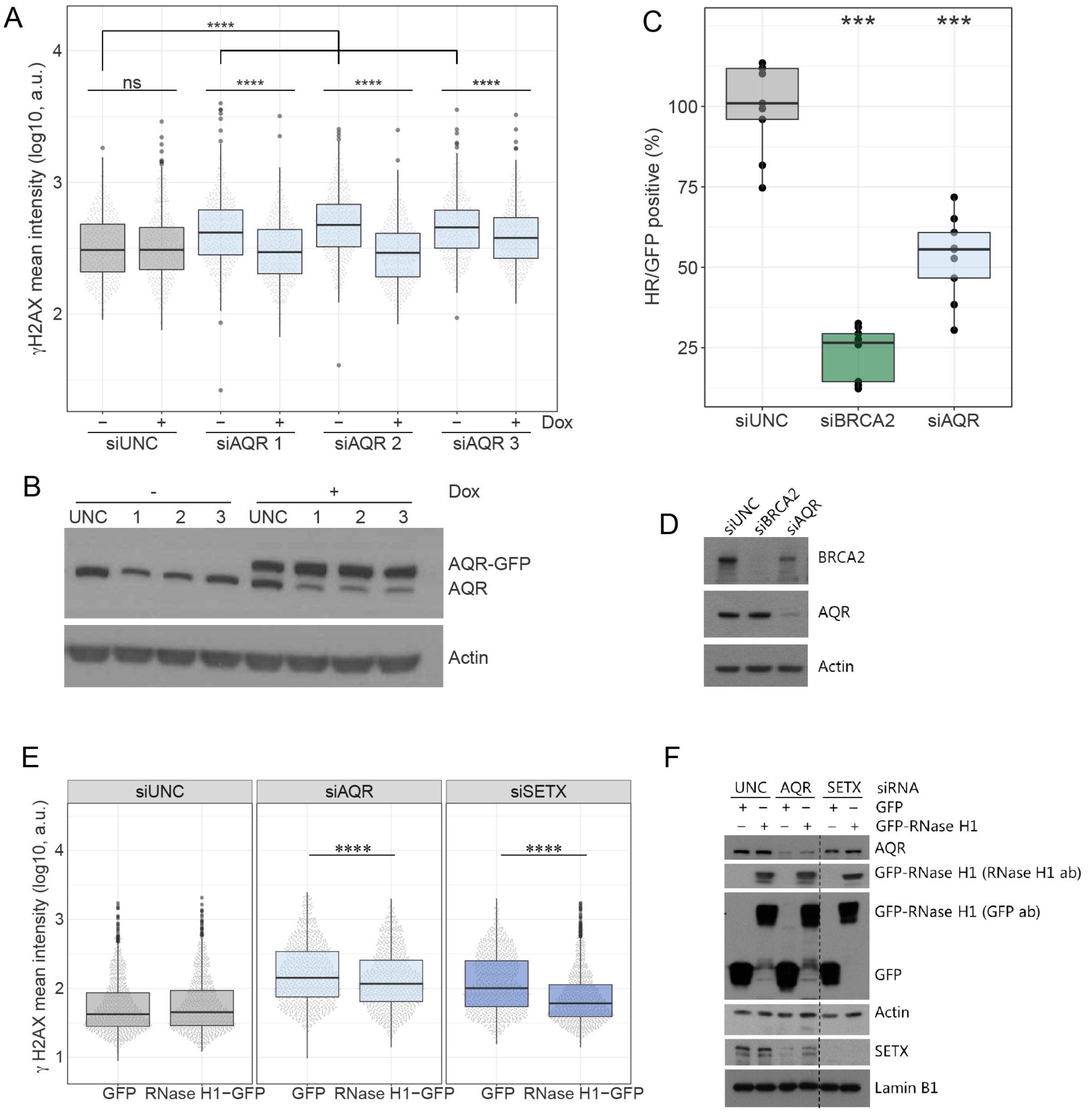

Fig. S8. ***AQR* suppresses genome instability**

A) Analysis of AQR re-expression on genome instability in MCF10A cells with Dox-inducible AQR-GFP expression. The y-axis corresponds to the γH2AX mean intensity. The numbers 1-3 represent cells transfected with three different AQR siRNAs. Significance levels were calculated for the comparison of all three AQR siRNAs with siUNC in the absence of AQR-GFP (-Dox) as well as comparing yH2AX levels in non-induced (-Dox) vs induced (+Dox) cells for each siRNA, **** P<0.0001.Representation of one out of three biological replicates, per sample n=450, n=150 per technical replicate. **** P<0.0001.

B) Immunoblot of samples in B showing AQR depletion as well as AQR-GFP expression. Actin was used as a loading control.

C) Analysis of HR-efficiency in AQR- or BRCA2-depleted U-2 OS cells using the DR-GFP reporter in U-2 OS cells. 48 h post siRNA-transfection cells were transfected with I-Sce1 plasmid and harvested 24 h later. The x-axis represents the HR efficiency in S and G2 cells normalised to the siUNC control. Nine biological replicates ($n=9$) are represented, p-value is calculated using Wilcoxon ranksum test. ***$P<0.001$.

D) Immunoblot of samples shown in D blotted using antibodies targeting AQR, BRCA2 and Actin, which was used as a loading control.

E) Analysis of γH2AX mean intensity following GFP or RNase H1-GFP expression (24 h) in AQR and SETX-depleted U-2 OS cells. γH2AX mean intensity in cells transfected with either AQR or SETX siRNA compared with siUNC control showed a significant increase in γH2AX mean intensity ($p<0.0001$). Representation of one out of three biological replicates, per sample $n=450$, $n=150$ per technical replicate. **** $P<0.0001$.

F) Immunoblot of samples shown in F evaluating AQR and SETX protein levels, and GFP and GFP-RNase H1 expression. Actin was used as a loading control for AQR and GFP blots, Lamin B1 was used as a loading control for the SETX blot.

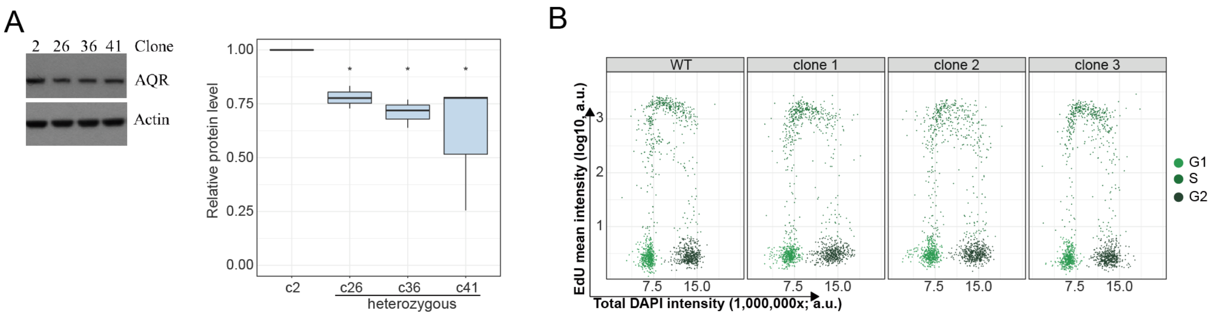

Fig. S9. Characterization of *AQR* heterozygous MCF10A iCas9 p53KO clones

A) Analysis of AQR protein levels by WB in AQR heterozygous MCF10A iCas9 p53KO clones, immunoblot and quantification, same samples as in Figure 4B. Actin served as a loading control. Analysis of three independent WB, * P< 0.05.

B) Cell cycle analysis of wild type or three heterozygous *AQR* MCF10A iCas9 p53KO clones. The x-axis corresponds to the total DAPI intensity and the y-axis to the EdU mean intensity. Cells were gated into G1, S and G2 phase based on total DAPI and mean EdU intensity. Representation of one out of three biological replicates, n=450 per sample, n=150 per technical replicate.

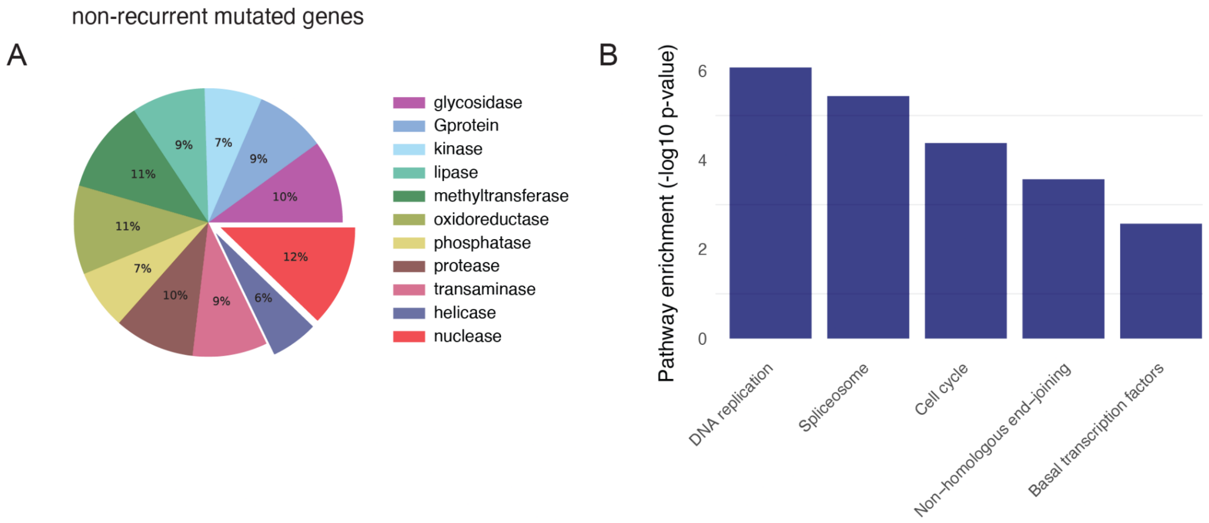

Fig. S10. Non-recurrent mutated genes

A) Non-recurrently mutated genes (less than 3 focal mutations, or a pan-cancer focal recurrence < 0.1%), essentiality (Dependency score < -1) and not found biallelic inactivated (<0.5%).

B) Significantly enriched KEGG pathways for Hemizygous driver genes. Only significant pathways are shown.

Table S1. (separate file)

Results of regression analysis assessing the effect of gene expression levels on mutation rates. The two sheets summarize regression statistics by enzyme family (Sheet 1) and by individual gene (Sheet 2) across various cancer types. Each row includes gene name, enzyme family, cancer type, effect size estimate, standard error, p-value, and Benjamini-Hochberg (BH) adjusted p-value. In the per-gene table, p-values are derived from linear regression (t-test) evaluating the association between gene expression and mutation rate.

Table S2. (separate file).

A collection of cancer-associated genes (GENE_NAME) from the Cancer Gene Census. Each gene is categorized by its enzyme class (ENZYME_CLASS), and annotated with relevant cancer hallmarks (HALLMARK).

Table S3. (separate file)

GISTIC peak enrichment analysis showing significant deletions and amplifications across enzyme families. Results are presented as combined p-values per enzyme family (Fisher's method, Sheet 1) and per cancer type (Fisher's exact test, Sheet 2).

Table S4. (separate file)

List of the genes used to prepare the siRNA libraries

Table S5. (separate file)

List of putative Hemizygous drivers.

Table S6. (separate file)

siRNA Abs primers sequences.
